# Taxonomic revision of the *Plagiothecium curvifolium* complex

**DOI:** 10.1371/journal.pone.0275665

**Published:** 2022-11-09

**Authors:** Grzegorz J. Wolski, Paulina Nowicka-Krawczyk, William R. Buck

**Affiliations:** 1 Faculty of Biology and Environmental Protection, Department of Geobotany and Plant Ecology, University of Lodz, Lodz, Poland; 2 Faculty of Biology and Environmental Protection, Department of Algology and Mycology, University of Lodz, Lodz, Poland; 3 Institute of Systematic Botany, The New York Botanical Garden, Bronx, NY, United States of America; Universite de Liege, BELGIUM

## Abstract

Supported by the examination of specimens from the entire range and by the analysis of type specimens and the diagnosis of individual names, morphological and genetic studies of the *Plagiothecium curvifolium* complex resulted in the conclusion that this taxon should be recognized as four separate taxa. In addition to *P*. *curvifolium* s.str., there is a variety that is proposed as a new combination–*P*. *curvifolium* var. *recurvum*; resurrection of the forgotten *P*. *decursivifolium*; and the description of a new species–*P*. *imbricatum*. The features that distinguish individual taxa focus primarily on: plant size; arrangement of leaves on the stem; the symmetry, dimensions, shape, concavity and folding of leaves; cell length; serration of the leaf apex; the shape of the decurrencies; the length of the sporophyte and the shape of the operculum. For all described taxa, the distribution, ecological preferences, key to their identification and detailed photographic documentation have been provided.

## Introduction

*Plagiothecium curvifolium* Schlieph. *ex* Limpr., a fairly widespread species in the Northern Hemisphere, is common in Europe, less frequentin North America and Asia, and considered doubtful in North Africa [1–3].

**Fig 1:**
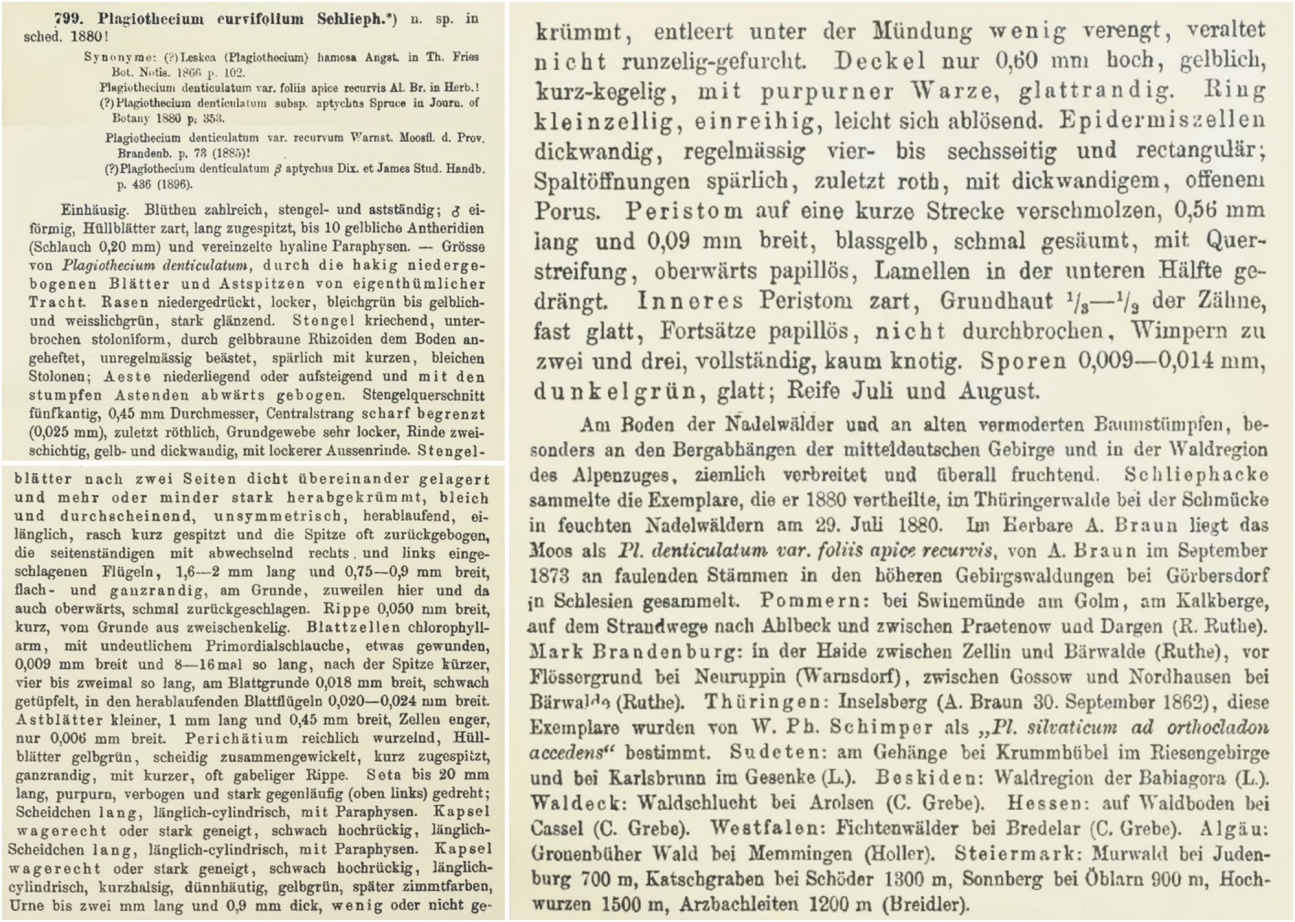
Diagnosis of *Plagiothecium curvifolium* [4].

This species was described 125 years ago in *Die Laubmoose Deutschlands*, *Oesterreichs und der Schweiz* [4]. Limpricht, in the diagnosis, does not indicate any specimen as a holotype. However, in the protologue (Fig 1) he indicated a Karl Schliephacke collection as the one on which this taxon was described: „Schliephacke sammelte die Exemplare, die er 1880 vertheilte, im Thüringerwalde bei der Schmücke in feuchten Nadelwäldern am 29. Juli 1880.” Limpricht [4] listed specimens from this collection in the diagnosis, along with other analyzed materials (Fig 1).

After publication, at the end of the 19^th^ and the beginning of the 20^th^ century, this species was not distinguished by researchers [e.g., 5–8]. On the other hand Meylan [9], shortly after its publication, proposed to change its status, recognizing it as a variety of *P*. *denticulatum*–*P*. *denticulatum* var. *curvifolium* (Schlieph. *ex* Limpr.) Meyl., additionally he proposed a distinctive form–*P*. *denticulatum* var. *curvifolium* fo. *albescens* Meyl.

At the turn of the 19^th^ and 20^th^ centuries, several varieties of *P*. *denticulatum* were published, which are considered as synonyms of *P*. *curvifolium sensu lato* [2,4,10,11]. For example, Dixon [12,13], citing Spruce [14], wrote about plants (at the rank of variety) characterized by downward curved leaves. He undoubtedly made a mistake, because Spruce [14] described this as a subspecies–*P*. *denticulatum* subsp. *aptychus* Spruce. Despite this error, the morphological features caused Dixon [10] to consider this taxon a synonym of *P*. *curvifolium*. On the other hand, Grout [15] proposed to change the status of this taxon to a variety–*P*. *denticulatum* var. *aptychus* (Spruce) Grout, stating that these plants, e.g., have”leaves smooth and shining, not shrunken (…) apices usually more or less curved downwards”, which strongly suggests plants currently understood as *P*. *curvifolium sensu lato*.

In the first half of the 20^th^ century *P*. *curvifolium* was distinguished in *Index Bryologicus* [16] and also was listed in central and northern Europe [17–24] and from North America [25,26]. During this period, a number of varieties and forms were described for *P*. *curvifolium*. For example: *P*. *curvifolium* var. *albescens* Warnst., which after 21 years Mönkemeyer [21] demoted it to form–*P*. *curvifolium* fo. *albescens* (Warnst.) Mönk., while after another 23 years Jedlička [24] proposed a new combination of this taxon considering it a separate species–*P*. *albescens* (Warnst.) Jedl.; *P*. *curvifolium* var. *subundulatum* Warnst., whose status has also been changed to form–*P*. *curvifolium* fo. *subundulatum* (Warnst.) Podp. *in* Jedl. [23]; or *P*. *curvifolium* var. *majus* Mönk. *in* Geheeb.

The next decades of the 20^th^ century saw many new forms of *P*. *curvifolium*, e.g., *P*. *curvifolium* fo. *julaceum* Culm. & E.Bauer; *P*. *curvifolium* fo. *gracile* A.Kopsch *ex* Jedl., for which Jedlička [23] also proposed a subform–*P*. *curvifolium* fo. *gracile* subfo. *propaguliferum* Jedl. Josef Jedlička [22–24], in his taxonomic studies, proposed many forms and subforms of this species (*P*. *curvifolium* fo. *latifolium* Jedl.; *P*. *curvifolium* fo. *longifolium* Jedl.; *P*. *curvifolium* fo. *phyllorhizans* Jedl.; *P*. *curvifolium* fo. *propaguliferum* Jedl.; *P*. *curvifolium* fo. *splendidum* Jedl.; *P*. *curvifolium* fo. *umbrosum* Jedl.; *P*. *curvifolium* fo. *gracile* Jedl. and *P*. *curvifolium* fo. *gracile* subfo. *propaguliferum*) however, none of them is currently recognized.

In the second half of the 20^th^ century, the understanding of *P*. *curvifolium* changed significantly, which was related to three important taxonomic revisions [27–29]. These studies strongly influenced the way this taxon was perceived by successive generations of bryologists, because it was proposed to abandon the recognition of any subspecies, varieties, forms and subforms of this taxon, or even its synonymy with *P*. *laetum* [27–29].

Ireland [27], in his revision of specimens originating in North America (Canada and U.S.A.) and subsequent articles [30,31], treated species of *Plagiothecium* very broadly. In addition, he proposed many synonyms, including *P*. *curvifolium* with *P*. *laetum*, which has led to a significant reduction in the number of taxa recognized in this genus from North America. This point of view was adopted and maintained in this area over the next decades [32,33], and it did not change until the beginning of the 21^st^ century [2,34].

Another revision based on material from Japan was done by Iwatsuki [28]. This author proposed a synonymization of the specimens described as *P*. *laetum* by Sakurai [35] with *P*. *curvifolium* and the exclusion of the former from the Japanese bryoflora. This point of view was adopted in subsequent studies by Japanese scientists [36,37]. But, on the other hand, Iwatsuki [28] mentioned that „*P*. *curvifolium* may be a variety of *P*. *laetum*” which is reflected in another Japanese moss checklist, where Iwatsuki [38] proposed to recognize *P*. *curvifolium* as a synonym *P*. *laetum*. So far, this approach has been recognized and accepted in Japan [39].

The third revision, proposed by Lewinsky [29], was of specimens from Denmark and has been widely adopted in Europe. In this publication, Lewinsky [29] disagreed with Ireland [27,30,31] and Iwatsuki [28,36,38] and distinguished both of the above-mentioned species. However, she pointed out that *P*. *curvifolium* is very variable and can sometimes be mistaken with *P*. *laetum* or even *P*. *denticulatum* [29].

Only the end of the 20^th^ century brought a taxonomic article proposing a new variation of the described species–*P*. *curvifolium* var. *hypnophyllum* Ukrainskaya [40]. However, this variety is currently not accepted [41–43], and recently only one form of this taxon is recognized–*P*. *curvifolium* fo. *julaceum* [2,3].

In the diagnosis of *P*. *curvifolium*, Limpricht [4] described in detail the new species, writing, e.g., that its turf is creeping, loose and clearly glossy; color is yellowish green to light green; leaves 1.6–2.0 × 0.75–0.9 mm, asymmetrical, ovate, overlapping, tightly arranged on the stem and more or less downwards; margin is entire; cells 9 μm wide and they are 8–16 times as long as they are wide; seta up to 2 cm; capsule horizontal or inclined.

On the other hand, accepted taxonomic studies and identification keys pointed out that stems of *P*. *curvifolium* are creeping [28,44,45]; turf are glossy, rather glossy to strongly glossy [29,37,44], pale green or yellowish-green to brownish-green [28,37,44,45]; leaves are ovate, oblong-ovate, oblong to lanceolate [28,37,24–46], symmetrical or almost symmetrical to asymmetrical [28,29,37,44–47]; leaves 1.0–2.6 × 0.5–0.9 mm [28,37,44,47]; margins are entire or with denticulations at apex [28,29,37,44–47]; costae are thin and short or long and strong [28,44–48]; cells from the middle part of the leaf are 80–160 × 6–10 μm [28,29,37,44–47]; seta are 8–30 mm long [28,29,37,44–47]; and capsules are horizontal, inclined, curved or not [37,45,46].

This summary shows that *P*. *curvifolium sensu lato* is recognized as very variable and sometimes difficult to distinguish, e.g., from the *P*. *laetum* complex or *P*. *denticulatum sensu lato* [28,37,45,46]. Additionally, as indicated by the above data, the range of variability of taxonomically significant features in relation to the features specified in the diagnosis is very wide [4,28,37,45,46]. This shows that this taxa is currently too broadly defined and may reflect a complex of taxa.

Taking into account the above facts, research was undertaken aimed at a taxonomic revision of *Plagiothecium curvifolium sensu lato* throughout its entire geographical range.

## Materials and methods

### Taxonomic analyses

During the conducted research specimens of *P*. *curvifolium sensu lato* from throughout its range from Asia, Europe and North America were revised (S1 Text). Specimens came from the: BM, C, F, HBG, JE, LOD, MO, PC, SZUB-B, UBC, VLA, WRSL, YU. The available types were analyzed: *P*. *curvifolium* (JE04004091), *P*. *curvifolium* fo. *julaceum* (C-M-9120, MO3974490), *P*. *curvifolium* var. *hypnophyllum* (VLA), *P*. *denticulatum* var. *recurvum* (HBG02115, HBG, JE04004201, PC01322640, WRSL), also the protologues of each name [4,9,14,15,18,21,23,24,40,49], as well as other types of this genus, such as *P*. *decursivifolium* Kindb. were analyzed (PC0132686).

Additionally, other specimens of *P*. *curvifolium* that had been previously genetically tested (2) were borrowed and examined (CP10515, CP10621) from the herbarium of C.

### DNA isolation, amplification and sequencing

Leafy stems of mosses were cut from dried material. Approximately 20 mg of dry tissue from each specimen in duplicates was placed in a 1.5 ml Eppendorf Safe-Lock tube and frozen (-20°C) for homogenization. Tissue homogenization was performed using a hand-held stainless steel homogenizer (Schlüter Biologie, Eutin, Germany). Total DNA was extracted using the GeneMATRIX Plant & Fungi DNA Purification Kit (Eurx, Gdansk, Poland) following the manufacturer’s protocol. DNA extracts were quantified with a BioDrop DUO Spectrophotometer (BioDrop Ltd, Cambridge, UK). From the duplicates, the sample with higher quality DNA (1.7–1.9 OD_260_/OD_280_) was selected for further analysis.

The molecular research was based on nuclear and chloroplast DNA markers: ITS (from the 3’ end of the hypervariable nuclear spacer ITS1, through the 5.8S gDNA, to the 5`end of the ITS2 spacer); and cpDNA genes: *trn*K*-psb*A (*mat*K) encoding maturase K, and *rpl*16 encoding ribosomal protein L16. Markers were selected based on Wynns et al. [50]; Ignatova et al. [51]; Wolski, Nowicka-Krawczyk [52] and Wolski et al. [3] *Plagiothecium-*focused studies.

For each sample, all markers were amplified by PCR in a few replicates to obtain high quality amplicons for sequencing. PCR for ITS and *rpl*16 was performed using primers and reaction conditions as described in Wolski, Nowicka-Krawczyk [52]. To obtain the best results in *trn*K-*psb*A (*mat*K) amplification, three parallel reactions were performed. Two reactions amplified the region in overlapping fragments using following set of primers: 1) trnK-F/matK-1307 and 2) trnk-450F/psbARbryo; while for some cases third reaction had to be performed to obtain high quality of a amplicon containing the *trn*K-*psb*A spacer using 3) trnK-2284F/psbARbryo set of primers. The reaction conditions for first two amplifications (1–2) were: 96°C (3 min); 53°C (1 min); 72°C (5 min); 41× [94°C (30 sec); 48°C (1 min); 72°C (4 min)] 72°C (20 min); while for the *trn*K-*psb*A spacer (3) 96°C (1.5 min); 51°C (1 min); 68°C (5 min); 41× [94°C (30 sec); 49°C (1 min); 68°C (4 min)] 68°C (20 min). Each reaction was performed in a 50 μl volume with 25 μl of Color Taq PCR Master Mix (2×) (Eurx, Gdansk, Poland).

PCR products were visualized on an agarose gel (1.5%, 90 V, 40 minutes) stained with GelRED^™^ fluorescent dye (Biotum, Fremont, CA, USA) and two replicates of each marker per sample were chosen for sequencing. Amplicons after PCR reaction were cleaned using Syngen Gel/PCR Mini Kit (Syngen Biotech, Wroclaw, Poland) according to the manufacturer’s protocol. Samples were sequenced with Sanger sequencing using primers from amplification by SEQme s.r.o. company (Dobris, Czech Republic). The obtained sequences were assembled in Geneious 11.1.5 (Biomatters Aps, Aarhus, Denmark) (http://www.geneious.com). The sequences were submitted to the NCBI GenBank database (www.ncbi.nlm.nih.gov) under the accession numbers ON202485-ON202493 for ITS, ON228316-ON228324 for *trn*K-*psb*A (*mat*K), and ON228307-ON228315 for *rpl*16.

### Phylogenetic analyses

Phylogenetic analyses of studied specimens and other species in the *Plagiothecium* group were performed based on concatenated ITS-*mat*K-*rpl*16 sequences matix (4127 bp). Voucher information for the specimens included in this study, with corresponding GenBank accession numbers, are presented Table 1. Sequences were aligned using the MAFFT v. 7 web server [53] (http://mafft.cbrc.jp/alignment/server/) where the auto strategy was applied, the scoring matrix of 200PAM with Gap opening penalty of 1.53, UniREf50 for Maft-homologs and Plot and alignment with threshold of 39 score were set. The obtained alignments were checked for poorly and ambiguously aligned regions and small corrections were made by eye. The evolutionary models were calculated using PartitionFinder 2 software [54] chosen according to the Akaike Information Criterion. Summary of partitions for ITS-*matK*-*rpl16* matrix evolutionary model selection and phylogenetic interference were submitted to figshare online database (10.6084/m9.figshare.16570353.v1).

**Table 1:** Voucher information and accession numbers for the specimens included in the phylogenetic analyses.

Taxon	Collection	Locality	ITS	matK	rpl16
*Isopterygiopsis pulchella*	UC barcode 1947397	USA: CA	KY550336	KY562830	KY514042
*Plagiothecium berggrenianum*	S-B44769	Russia: Pacific Siberia, Yakutiya	KY550267	KY562760	KY513972
*Plagiothecium brasiliense*	E barcode E00387968	Brazil	KY550266	KY562759	KY513971
*Plagiothecium conostegium*	NY: *S*.*P*. *Churchill et al*. *19839*	Bolivia	KY550271	KY562764	KY513976
*Plagiothecium conostegium*	NY barcode 00845279	Guatemala	KY550318	KY562812	KY514024
*Plagiothecium conostegium*	S-B53327	Mexico	KY550272	KY562765	KY513977
*Plagiothecium curvifolium*	DUKE barcode 0209096	Canada: BC	KY550273	KY562766	KY513978
*Plagiothecium curvifolium*	CP: *G*.*P*. *Rothero s*. *n*.	Germany: Hochschwarzwald	KF882228	KF882128	KF882328
*Plagiothecium curvifolium*	CP: *J*.*T*. *Wynns 1939*	Denmark: Kongelunden, Amager	KF882227	KF882127	KF882327
*Plagiothecium denticulatum*	CP: *J*.*T*. *Wynns 2081*	Denmark: Soroe kommune, Sjaelland	KF882229	KF882129	KF882329
*Plagiothecium denticulatum*	BONN: *O*.*M*. *Afonina s*.*n*.	Russia: Far East, Chukotka	KY550275	KY562768	KY513980
*Plagiothecium denticulatum*	C: *R*.*R*. *Ireland 23098*	Canada: ON	KY550276	KY562769	KY513981
*Plagiothecium denticulatum* var. *bullulae*	UC barcode 1798690	USA: NV	KY550278	KY562771	KY513983
*Plagiothecium denticulatum* var. *bullulae*	UC barcode 1947417	USA: CA	KY550277	KY562770	KY513982
*Plagiothecium denticulatum* var. *obtusifolium*	CP: *J*.*T*. *Wynns 2842*	Germany: Schauinsland, Hochschwarzwald	KF882230	KF882130	KF882330
*Plagiothecium denticulatum* var. *obtusifolium*	UC barcode 1724036	USA: WA	KY550279	KY562772	KY513984
*Plagiothecium denticulatum* var. *pungens*	DUKE barcode 0150010	USA: AK	KY550280	KY562773	KY513985
*Plagiothecium laetum*	CP: *J*.*T*. *Wynns 2907*	Germany: Schauinsland, Hochschwarzwald	KF882234	KF882134	KF882334
*Plagiothecium laetum*	C barcode CP0010626	USA: NC	KY550292	KY562785	KY513997
*Plagiothecium laetum*	C barcode CP0010627	USA: NC	KY550293	KY562786	KY513998
*Plagiothecium lamprostachys*	S-B54613	Australia: VIC	KY550284	KY562777	KY513989
*Plagiothecium lamprostachys*	DUKE barcode 0156846	Australia: VIC	KY550285	KY562778	KY513990
*Plagiothecium lamprostachys*	S: *H*. *Streimann 47719*	Australia: NSW	KY550282	KY562775	KY513987
*Plagiothecium latebricola*	CP: *I*.*L*. *Goldberg s*. *n*.	Denmark: Holmegaards Mose, Sjaelland	KF882235	KF882135	KF882235
*Plagiothecium lucidum*	NY barcode 01233548	Chile	KY550298	KY562791	KY514003
*Plagiothecium lucidum* (*P*. *funale*)	BONN: *J*.*-P*. *Frahm 12–6*	New Zealand	KY550299	KY562792	KY514004
*Plagiothecium membranosulum*	BONN: *J*.*-P*. *Frahm 7756*	Democratic Republic of the Congo	KY550310	KY562803	KY514015
*Plagiothecium membranosulum*	S-B78514	South Africa	KY550303	KY562796	KY514008
*Plagiothecium membranosulum*	DUKE barcode 0016754	South Africa	KY550304	KY562797	KY514009
*Plagiothecium mollicaule*	NY barcode 1596265	Brazil	KY550300	KY562793	KY514005
*Plagiothecium ovalifolium*	DUKE barcode 0188886	Chile	KY550314	KY562807	KY514019
*Plagiothecium pacificum*	UC barcode 1921143	USA: CA	KY550295	KY562788	KY514000
*Plagiothecium platyphyllum*	C: *J*. *Lewinsky et al*. *s*. *n*.	Finland: Haluna, Nilsiae, Savonia borealis	KF882241	KF882141	KF882341
*Plagiothecium ruthei*	CP: *J*.*T*. *Wynns 1997*	Denmark: Lyngby Aamose, Sjaelland	KF882242	KF882142	KF882342
*Plagiothecium svalbardense*	C-M-9109	Greenland: W5	KY550296	KY562789	KY514001

Phylogenetic calculations were performed using maximum likelihood analysis (ML) in the IQ-TREE web server [55] (http://iqtree.cibiv.univie.ac.at/) with the ultrafast bootstrap (UFBoot) pseudolikelyhood algoritm [56] and 10000 replicates; and Bayesian inference (BI) in MrBayes 3.2.2 [57] where two parallel Markov chain Monte Carlo (MCMC) runs for four million generations each, with trees sampled every 1000 generations were performed. The average standard deviation of split frequencies in both cases remained below 0.01 for the last 1000 generations and posterior probabilities were estimated from the 50% majority-rule consensus tree after elimination of the first 25% of samples as burn-in. Raw data sequences, the alignment file, evolutionary model set for partitions and tree files were submitted to figshare online database (10.6084/m9.figshare.16570353).

## Results

Studied specimens (*P*. *curvifolium* JE04004091, HBG02115, PC01322640, WRSL; *P*. *curvifolium* fo. *julaceum* C-M-9120, MO3974490; *P*. *denticulatum* var. *recurvum* JE04004201), all analyzed names [4,9,14,15,18,21,23,24,40,49] as well as test specimens from the entire range of the studied taxon (C, F, HBG, JE, LOD, MO, PC, SZUB-B, UBC, VLA, WRSL, YU) are different from each other in terms of macro- and microscopic qualitative and quantitative characteristics. The most important of them differentiating individual taxa include, e.g., the size and color of the turf; arrangement of leaves on the stem; symmetry, dimensions and shape of the leaves; cell length; serration of the apex; the shape of the decurrencies; the length of the sporophyte and the shape of the operculum. This changeability is reflected in the genetic variability of the studied complex.

### Genetic analyses

**Fig 2:**
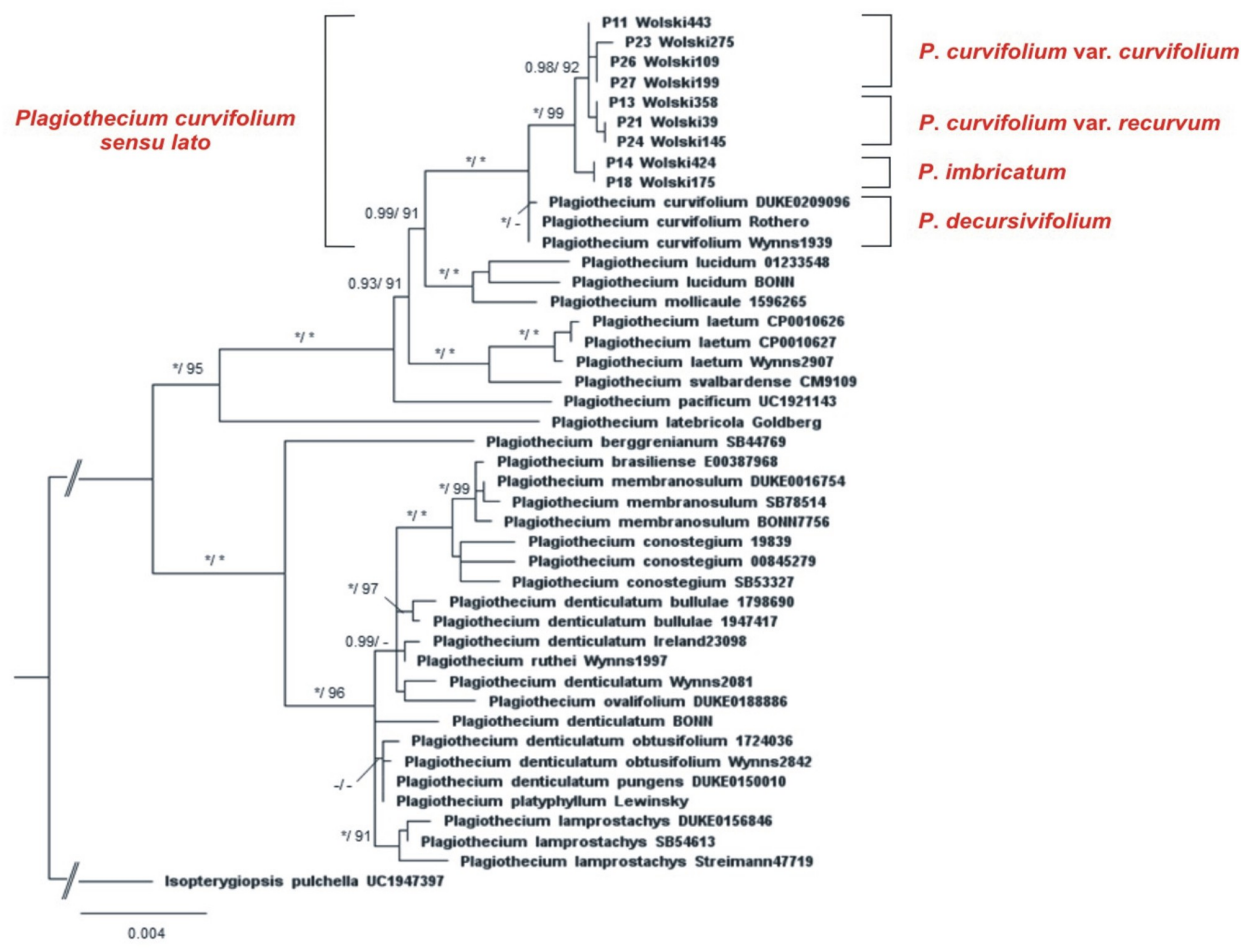
Phylogenetic tree of *Plagiothecium* taxa with *Isopterygiopsis pulchella* as the outgroup taxa based on concatenated nuclear (ITS1-5.8S-ITS2) and chloroplast (*mat*K and *rpl*16) DNA markers (total 4127 bp). The tree presents the position of Wolski morphotypes of *Plagiothecium* among the *Plagiothecium* group. Numbers on branches indicate posterior probabilities from BI analysis followed by bootstrap values from ML. Asterisk (*) indicates 1.00 (BI) and 100 (ML), while minus (-) indicates values below 0.9 (BI) and 90 (ML). The topology of the tree was based on ML analysis.

Phylogenetic analyses (Fig 2) based on the concatenated ITS*-mat*K-*rpl*16 placed studied specimens within the branch of a *Leptophyllum* sect. clade, next to “*P*. *curvifolium*” analyzed by Wynns [2], with a very high branch support by Bayesian inference (PP = 1) and maximum likelihood (B = 100). Moreover, with high branch support from both analyses (PP = 1; B = 99) the topology of the tree revealed division of the *Wolski* specimen clade into two subclades containing different morphotypes. As the first subclade was monospecific, the second possessed internal division highly supported by BI (PP = 0.98) (Fig 2).

### Taxonomic implications

High morphological variability of *P*. *curvifolium sensu lato* was reflected in the variability of the genetic material of individual specimens. All genetically tested samples: *Wolski 443* (LOD 15007), *Wolski 275* (LOD 15008), *Wolski 109* (LOD 15009), *Wolski 199* (LOD 15010), *Wolski 358* (LOD 15011), *Wolski 39* (LOD 15012), *Wolski 145* (LOD 15013), *Wolski 175* (LOD 15014) and *Wolski 424* (LOD 15015) were outside the clade „*P*. *curvifolium*” (Fig 2) analyzed by Wynns [2].

**Fig 3:**
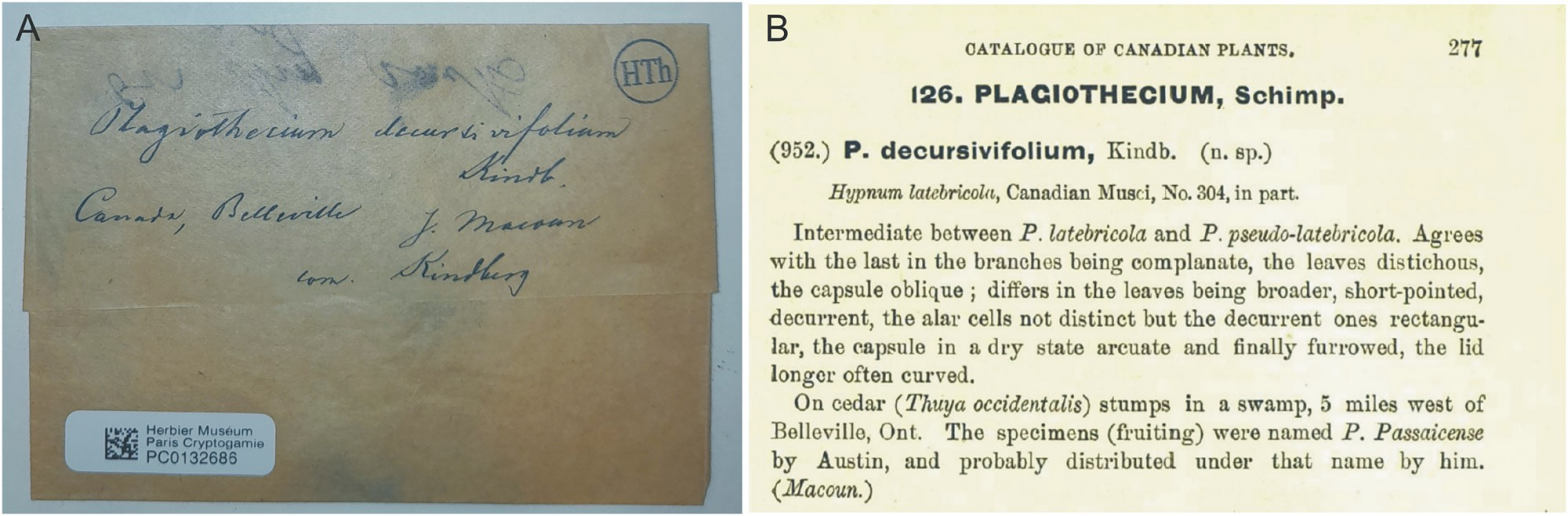
Specimen PC0132686 (A) lectotype of *P*. *decursivifolium* and diagnosis (B) of this name [58].

During this revision the type of *P*. *decursivifolium* (PC0132686), deposited at Herbarium PC, was tested. Its quite modest description on the envelope in part matches the diagnosis (Fig 3). The revision of this material showed that it clearly belongs to the *P*. *curvifolium* complex, also this is confirmed by the diagnosis [58]. Morphologically, it fits to the specimens (CP0010515, CP0010621) genetically examined by Wynns [2] and also is identical to the *P*. *curvifolium* fo. *julaceum* type (C-M-9120, MO3974490). All the above-mentioned specimens are characterized by, e.g., asymmetric, lanceolate, longitudinally folded, clearly concave, with a wide base and often torn leaves; decurencies usually forming very distinct auricles; capsules inclined to horizontal; and operculum rostrate.

Taking into account the above facts and accordance to Art. 9.3 of the *Shenzhen Code* [59], “A lectotype is one specimen or illustration designated from the original material (Art. 9.4) as the nomenclatural type, in conformity with Art. 9.11 and 9.12, (…)” specimen PC0132686 from Herbarium PC should be designated as the lectotype of *P*. *decursivifolium*.

Whereas, in the *P*. *curvifolium* fo. *julaceum* case taking into account the existence of two original materials of this taxon and on the basis of Art. 9.6 of the *Shenzhen Code* [59] “A syntype is any specimen cited in the protologue when there is no holotype, or any one of two or more specimens simultaneously designated in the protologue as types” all the above-mentioned specimens should be regarded as syntypes. Additionally, accordance to Art. 9.3 of the *Shenzhen Code* [59] quoted above specimen C-M-9120 from Herbarium C should be designated as the lectotype of *P*. *curvifolium* fo. *julaceum*.

#### Tested samples

*Wolski 443* (LOD 15007), *Wolski 275* (LOD 15008), *Wolski 109* (LOD 15009), *Wolski 199* (LOD 15010), *Wolski 358* (LOD 15011), *Wolski 39* (LOD 15012), and *Wolski 145* (LOD 15013) (Fig 2) form an internally genetically diverse clade. Specimens: *Wolski 443*, *Wolski 275*, *Wolski 109*, and *Wolski 199* represent the material with a fairly flat turf with leaves slightly curved towards the ground; leaves that are symmetrical or almost symmetrical, lanceolate, concave and very often incurved; decurrencies not forming distinct auricles; capsules inclined to horizontal; and an operculum conical, obtuse. These specimens morphologically correspond to the type of *P*. *curvifolium* (JE04004091, HBG02115, PC01322640, WRSL). While, the specimens: *Wolski 358*, *Wolski 39*, and *Wolski 145* represent the material, among others, with a turf with leaves strongly curved towards the ground; leaves that are asymmetrical, hooked, concave or slightly concave, very often folded; decurrencies not forming distinct auricles; capsules inclined to horizontal, and operculum rostellate, and these specimens fit perfectly to the *P*. *denticulatum* var. *recurvum* type (JE04004201) and other specimens like *P*. *curvifolium* var. *hypnophyllum* (VLA) type.

**Fig 4:**
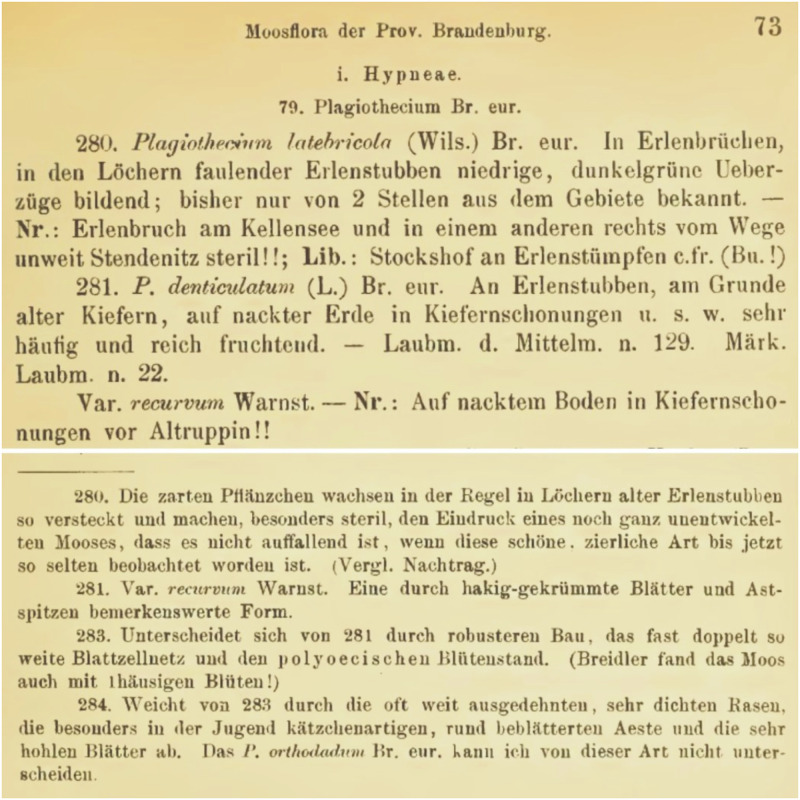
Diagnosis of *P*. *denticulatum* var. *recurvum* [60], modified.

**Fig 5:**
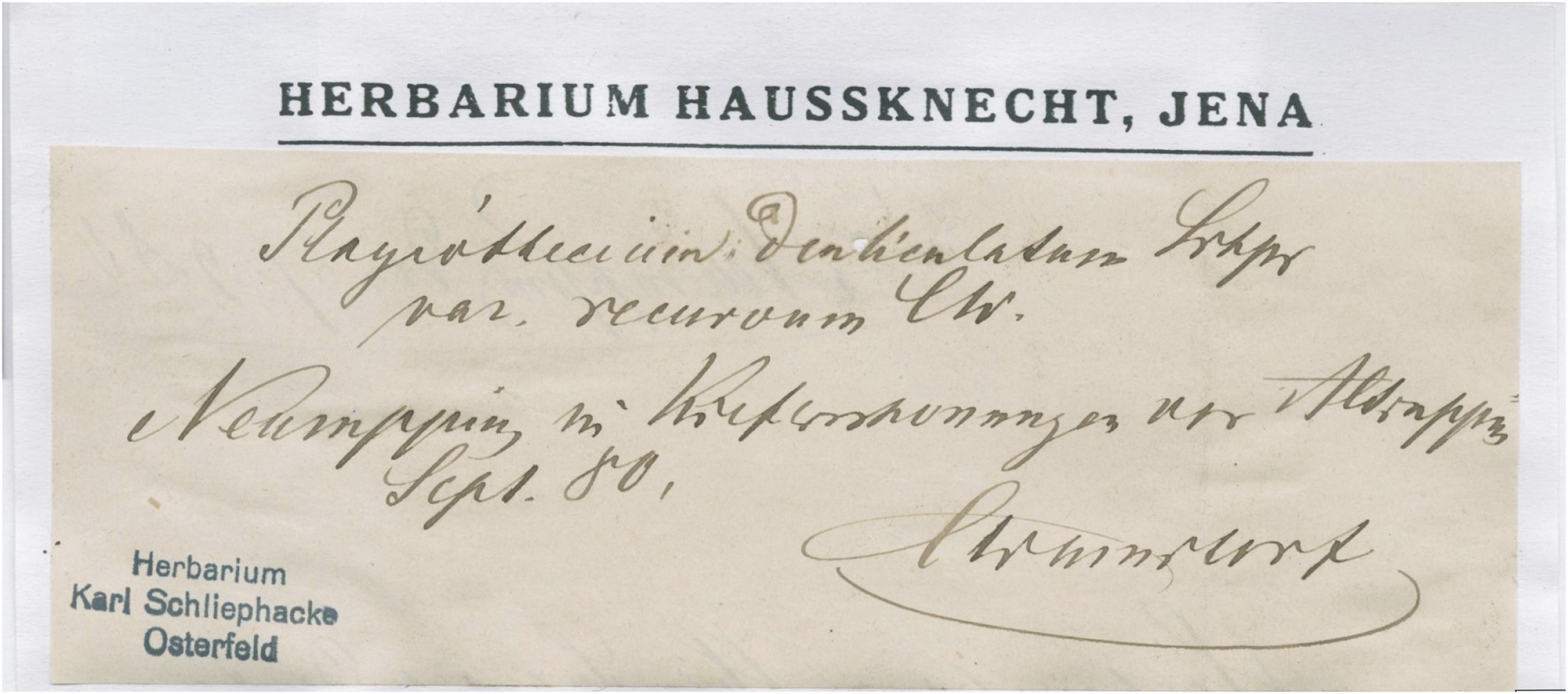
Specimen JE04004201 form JE Herbarium, lectotype of *P*. *denticulatum* var. *recurvum*.

Warnstorf [60], when proposing a new variety, *P*. *denticulatum* var. *recurvum*, described it as being distinguished by its hooked leaves. However, he did not indicate any original materials or types. He only wrote that „Auf nacktem Boden in Kiefernschonungen vor Altruppin!!” (Fig 4). During this revision, a specimen (JE04004201) was found in Herbarium JE, which, as stated on the envelope, was collected in September 1880 and signed by C. Warnstorf as „*P*. *denticulatum* var. *recurvum*” (Fig 5). Taking into account the above facts and that the location and habitat description is consistent with the diagnosis, specimen JE04004201 should be considered as original material on the basis of which Warnstorf [60] described the new variety. Taking into account the above facts and in accordance to Art. 9.3 of the *Shenzhen Code* [59], quoted above specimen JE04004201 form Herbarium JE should be designated as the lectotype of *P*. *denticulatum* var. *recurvum*.

When Limpricht [4] described *P*. *curvifolium* he proposed the synonymization of *P*. *denticulatum* var. *recurvum* with this species. In the diagnosis, the author did not indicate any specimen or figures as a type, but stated that the new species was described on the basis of materials collected in July 29, 1880 by Karl Schliephacke in damp coniferous forests in Thuringia near the town of Schmücke [4]. In the same year, according to the author, these specimens were sent to many herbaria (Fig 4).

**Fig 6:**
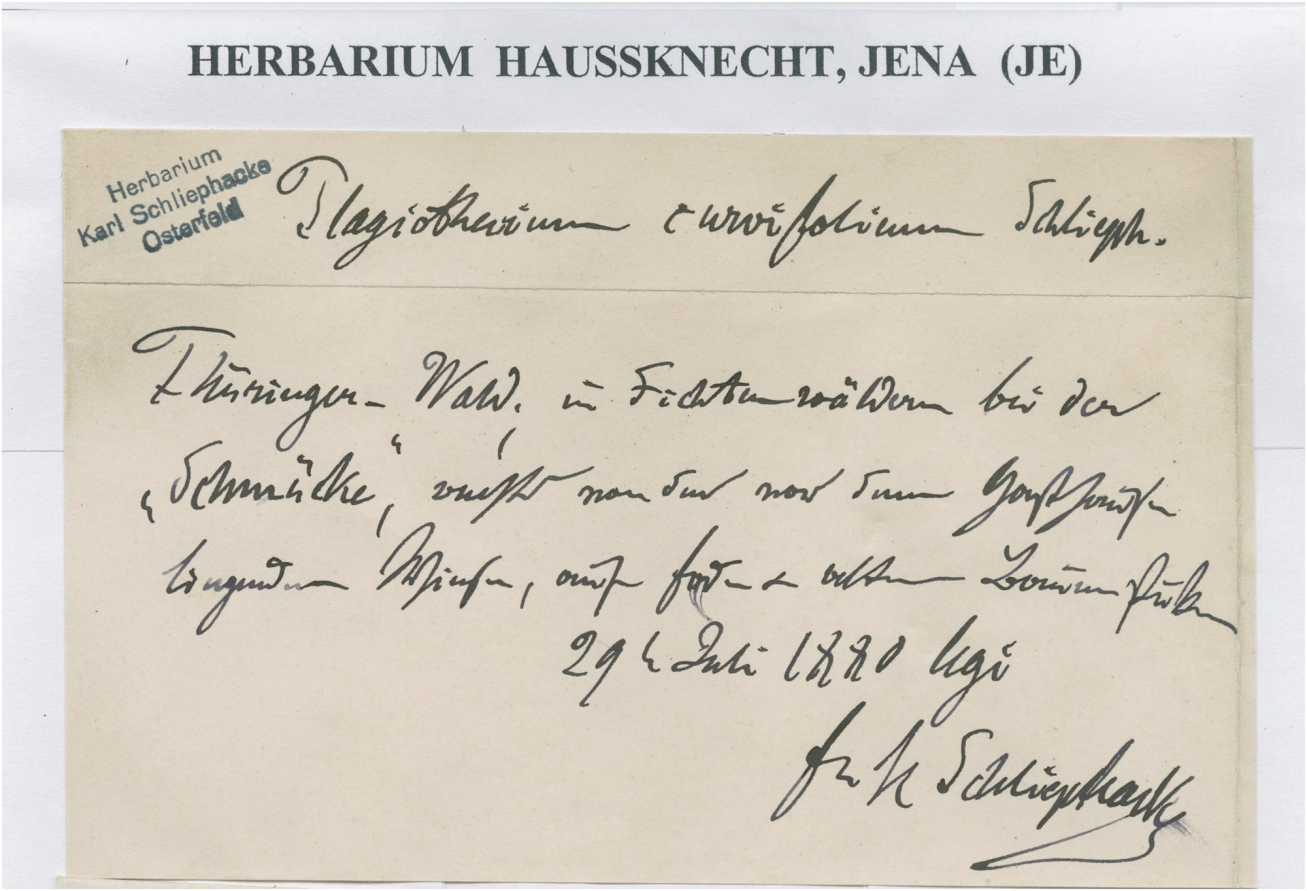
Specimen JE04004091 form JE Herbarium–lectotype of *P*. *curvifolium*.

**Fig 7:**
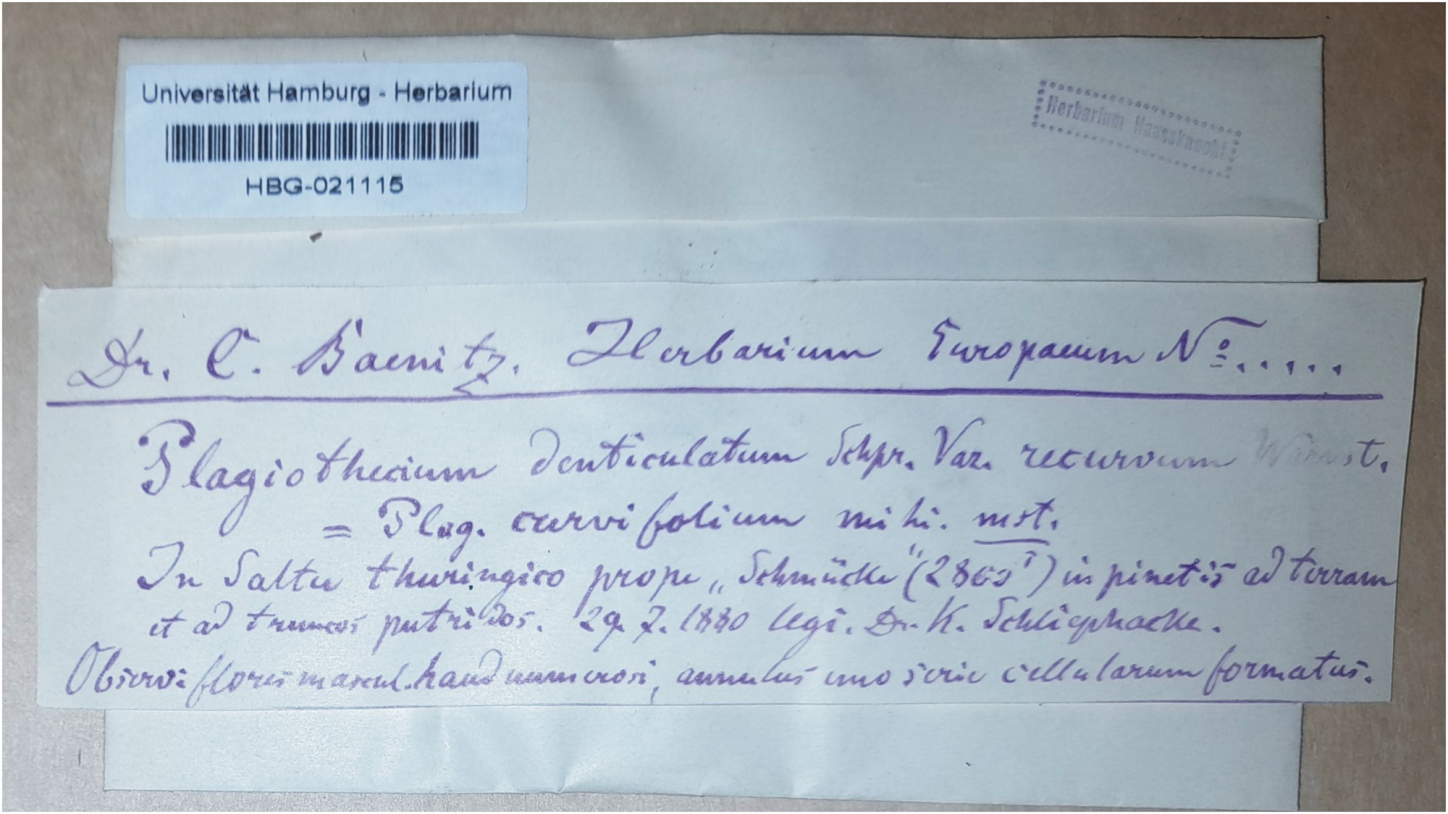
Material from the original Karl Schliephacke collection, one of many syntypes of *P*. *curvifolium* (Herbarium HBG).

During this revision, in Herbarium JE, a specimen (JE04004091) was found which was collected July 29, 1880 by C. Schliephacke and signed as „*Plagiothecium curvifolium* Schlieph.” (Fig 6). At the same time, in many European herbaria (e.g., HBG02115, PC01322640, WRSL) materials originating from *Herbarium Europeanum Dr Karl Baenitz* are deposited and labeled as *P*. *denticulatum* var. *recurvum* = *P*. *curvifolium* (Fig 7). Data on the envelopes of all the above-mentioned specimens indicating, e.g., Karl Schliephacke as their collector; date (July 29, 1880) and place of collection are consistent with the diagnosis of *P*. *curvifolium* [4], which indicates that they came from one collection and are the original materials used by Limpricht to describe a new taxon. Taking into account the above facts and on the basis of Art. 9.6 of the *Shenzhen Code* [59] quoted above all the above-mentioned Karl Schliephacke specimens should be regarded as syntypes.

All mentioned specimens are described quite similarly and contain data characterizing the Schliephacke collections [4], however with the difference that specimens JE04004091 (as diagnosis) is described in German, it contains the seal *’Herbarium Karl Schliephacke Osterfeld’* and is not as detailed as the other materials, but at the same time it more accurately reflects the data contained in the diagnosis. Whereas, the remaining materials are described in identical handwriting in Latin. Considering the above facts, and that specimen (JE04004091) has a large well-preserved turf of material, and according to Art. 9.3 of the *Shenzhen Code* [59] cited above specimen JE04004091 form Herbarium JE should be designated as the lectotype of *P*. *curvifolium*.

Genetic analyzes also show the third clade, which is composed of two specimens: *Wolski 175* (LOD 15014) and *Wolski 424* (LOD 15015) (Fig 2), these materials represent a new species–*P*. *imbricatum*, which, compared to the whole *P*. *curvifolium* complex, is characterized by a unique combination of gametophytic features.

Thus, the revision of herbarium materials, supported by the examination of types and original collections; a detailed analysis of the diagnoses of individual names; and the history of the described taxon supported by DNA research allowed four separate taxa within *P*. *curvifolium sensu lato* to be distinguished. Two of them are *P*. *curvifolium* and a variety of it which is proposed as a new combination; one is the resurrected *P*. *decursivifolium*; and the fourth is a newly described species–*P*. *imbricatum*.

### Description of individual taxa

***Plagiothecium curvifolium* var. *curvifolium*** Schlieph. *ex* Limpr.

Die Laubmoose Deutschlands, Oesterreichs und der Schweiz 3: 269. 1897.

***Lectotype*** (designated here): Germany, Thuringia, in feuchten Nadelwäldern, Schmücke, 29 July 1880, *D*. *K*. *Schliephacke* (JE04004091!, isolectotypes: HBG02115!, PC01322640!, WRSL! DUKE155945).

**Fig 8:**
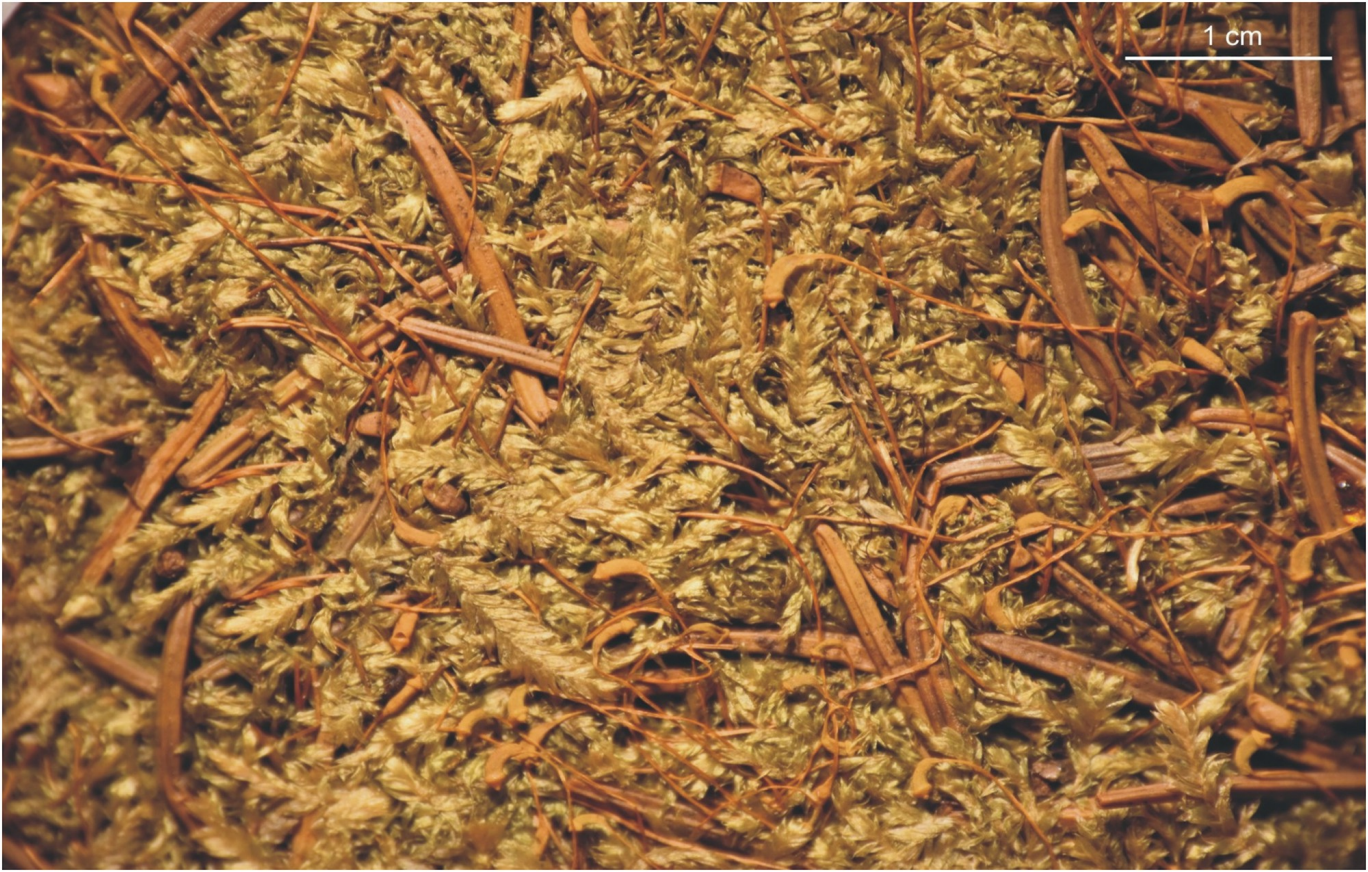
The turf of *Plagiothecium curvifolium* var. *curvifolium* with sporophytes (from the lectotype *Plagiothecium curvifolium*, *K*. *Schliephacke*, JE04004091), photo. G. J. Wolski, 11 September 2021.

**Fig 9:**
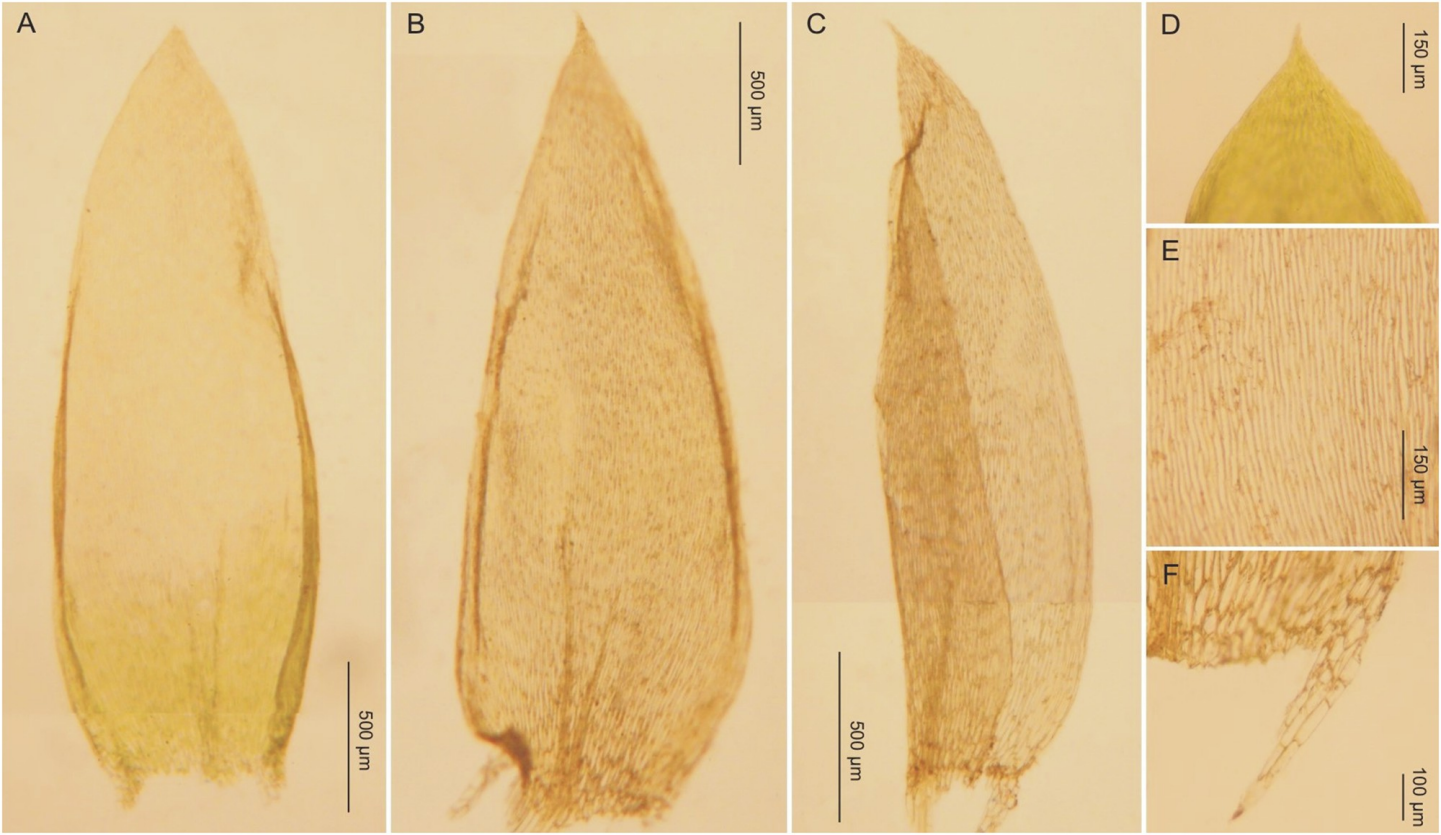
The most important taxonomic features of *Plagiothecium curvifolium* var. *curvifolium*. A–C—stem leaves (A—*G*. *J*. *Wolski*, *Wolski 199*, LOD 15010; B–C—from lectotype, *K*. *Schliephacke*, JE04004091); D—leaf apex (*G*. *J*. *Wolski*, *Wolski 275*, LOD 15008); E—cells from the middle part of the leaf (from lectotype, *K*. *Schliephacke*, JE04004091); F—decurrencies (from lectotype, *K*. *Schliephacke*, JE04004091), photo. G. J. Wolski, 11 September 2021.

***Description***: Plants medium-sized, yellow-green to green, with a metallic luster (Fig 8); stems complanate-foliate, gently imbricate, 1.5–2.5 cm long; leaves lanceolate to ovate-lanceolate, concave, slightly curved towards the ground. Symmetrical or almost symmetrical leaves dominating, those from the middle of the stem 1.7–2.7 (M 2.2) mm long, and the width measured at the widest point 0.7–1.5 (M 1.0) mm; margin incurved, delicately on both sides or strongly on one side; the apex acuminate, usually not denticulate; costae two, variable, but usually strong, usually to 1/3 leaf length, reaching 0.4–0.7 (M 0.5) mm; cells from the midleaf linear-vermicular, 110–155 (M 130) × 8–9 μm, areolation tight; decurrencies 250–370 (M 310) × 60–95 (M 62) μm, wedge-shaped, not forming distinct auricles, created by 2–3 rows of rectangular cells, 70–60 × 22–27 μm, some cells from external row inflated (Fig 9); seta 1.2–1.5 cm long; capsules inclined to horizontal, cylindrical, curved, 2.2–2.3 × 1.0 cm; operculum conical, obtuse, 625–650 μm long.

***Distribution and ecology***: the presently known range of *Plagiothecium curvifolium* var. *curvifolium* is Europe, Asia and North America. In this area, it is recorded mainly from coniferous forests (dominated by *Pinus*, *Picea* or *Abies*), less often from deciduous forests (dominated by *Fagus* or *Quercus*). This taxon most often is found on epigeic habitats (on soil, humus), less often is it epiphytic (bark of *Pinus*, *Picea*, *Fagus*, *Quercus*, *Alnus*), epixylic (logs) or epilithic (sandstones) (S1 Text).

***Additional specimens examined*:** as a supplementary materials (S1 Text).

***Plagiothecium curvifolium* var. *recurvum*** (Warnst.) G.J.Wolski & W.R.Buck, comb. nov.

*Plagiothecium denticulatum* var. *recurvum* Warnst., Verhandlungen des Botanischen Vereins für die Provinz Brandenburg und die Angrenzenden Länder 27: 73. 1885.

***Lectotype*** (designated here): Germany, prov. Brandenburg, auf nacktem Bodem in Kiefernschonungen vor Altruppin, Neuruppin, *C*. *Warnstorf* (JE04004201!).

*Plagiothecium curvifolium* var. *hypnophyllum* Ukrainskaya, *Novosti Sistematiki Nizaikh Rastenii* 31: *183*, f. 12–14. 1996, syn. nov.

***Type***: Prov. Mosquensis, distr. Krasnogorskensis, 2 km ad austro-occidentem a Krasnogorsk. Ad Betulam in silva 28 VII 1986, *Ignatov*. In herbario bryologico Horti Botanici Publici Mosquae (MHA, VLA) conservatur (*n*.*v*.).

**Fig 10:**
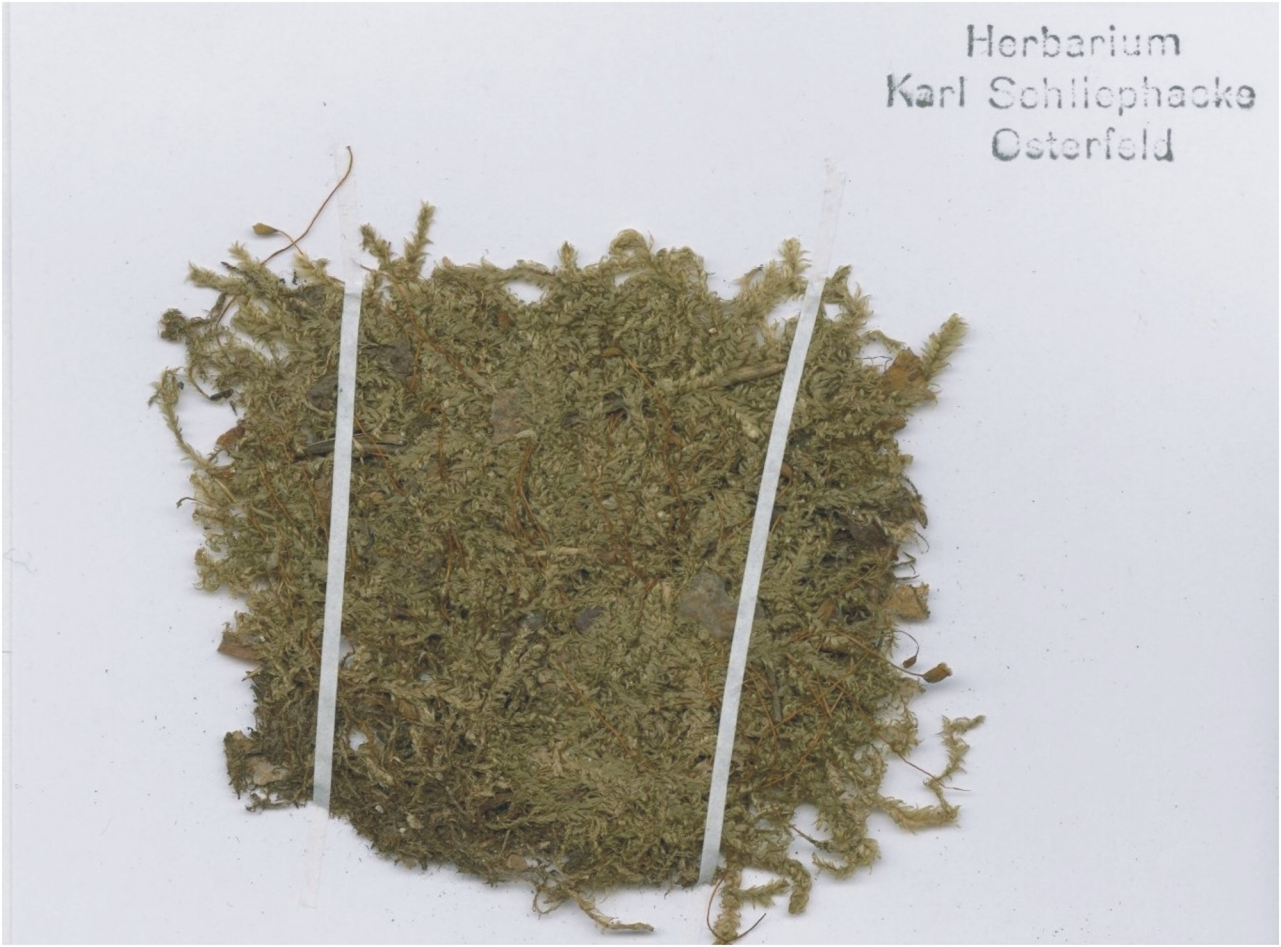
The turf of *Plagiothecium curvifolium* var. *recurvum* with sporophytes (from the lectotype, *Plagiothecium denticulatum* var. *recurvum*, *C*. *Warnstorf*, JE04004201) photo. G. J. Wolski, 12 September 2021).

**Fig 11:**
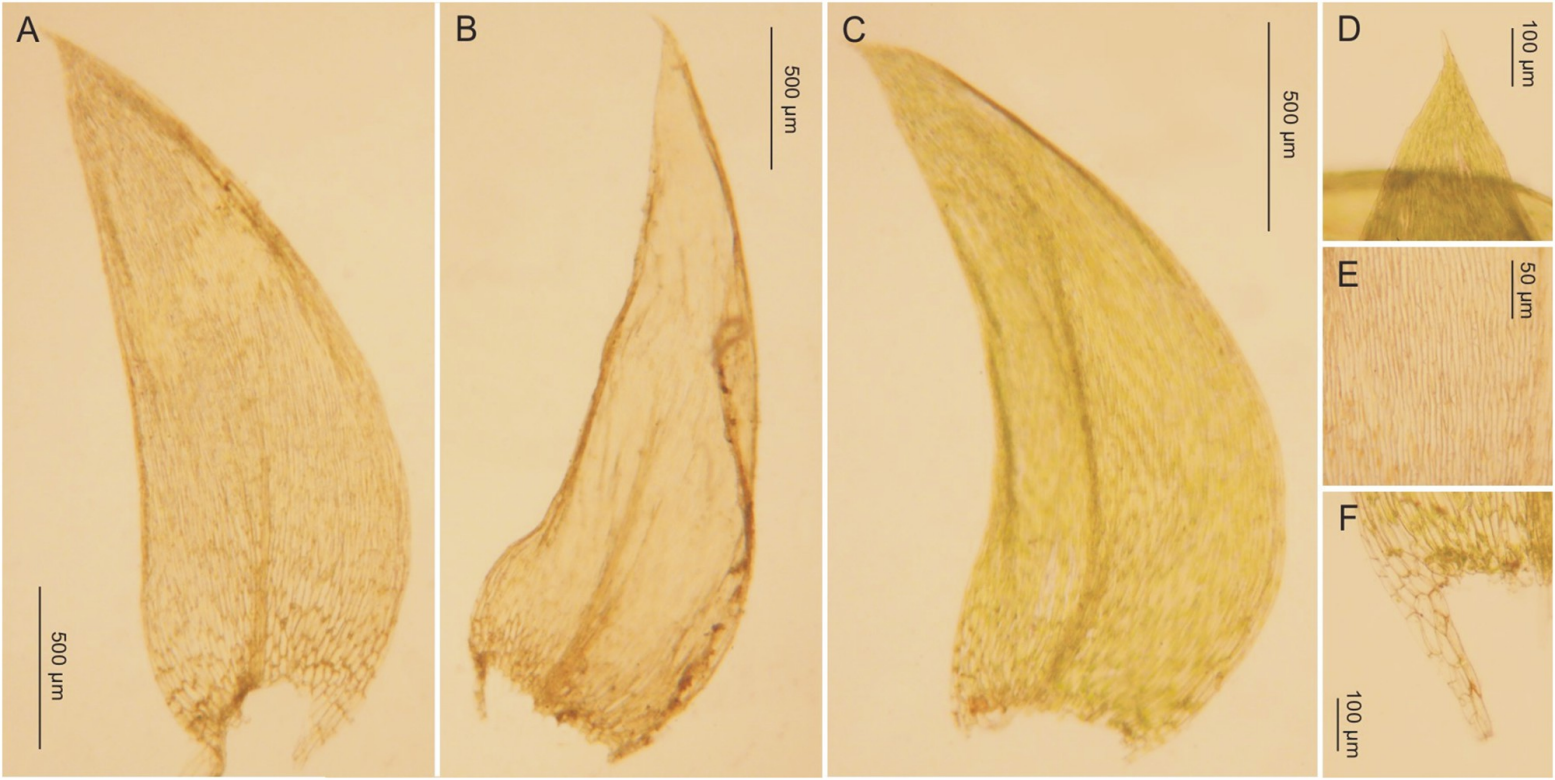
The most important taxonomic features of *Plagiothecium curvifolium* var. *recurvum*. A–C—stem leaves (A-B—from the lectotype, *C*. *Warnstorf*, JE04004201; C—*G*. *J*. *Wolski*, *Wolski 145* LOD 15013); D—leaf apex (*G*. *J*. *Wolski*, *Wolski 358* LOD 15011); E—cells from the middle part of the leaf (from lectotype, *C*. *Warnstorf*, JE04004201); F—decurrencies (*G*. *J*. *Wolski*, *Wolski 145* LOD 15013), photo. G. J. Wolski, 12 September 2021.

***Description***: Plants medium-sized, bright-green to green, with a metallic luster (Fig 10); stems complanate-foliate, 1.5–2.0 cm long; leaves lanceolate, concave, clearly curved towards the ground and sometimes clearly transversely folded when dry. Strongly asymmetrical, hooked leaves dominating, those from the middle of the stem 1.7–2.2 (M 2.0) mm long, and the width measured at the widest point 0.6–0.9 (M 0.750) mm; margin sometimes incurved; the apex acuminate, usually denticulate by 2–3 teeth; costae two, variable, but usually strong, extending ½ leaf length, reaching 0.3–0.7 (M 0.5) mm; cells from the midleaf linear-vermicular, 60–120 (M 100) × 7–9 μm, areolation tight; decurrencies 260–330 (M 295) × 90–100 μm, wedge-shaped, not forming distinct auricles, created by 2–3 rows of rectangular, sometimes inflated cells (Fig 11), 60–70 × 26–30 μm; sporophytes 1.7–2.5 cm long, capsules inclined, cylindrical, 1.8–2.2 × 0.7–0.9 cm; operculum rostellate.

***Distribution and ecology***: the currently known range of this taxon are Europe, Asia and North America. In this area it is mainly recorded from coniferous forests (mainly dominated by *Picea*, less often by *Pinus*, *Abies* or *Pseudotsuga*). Also, most often *P*. *curvifolium* var. *recurvum* is found in epigeic habitats (on mineral soil, humus, litter), less often is it epiphytic (bark of *Picea* or *Quercus*, *Fagus*), epixylic (log, stump) or epilithic (rock) (S1 Text).

***Additional specimens examined***: as a supplementary materials (S1 Text).

***Plagiothecium decursivifolium*** Kindb. *in* Macoun & Kindb., Catalogue of Canadian Plants, Part VI, Musci 277. 1892.

***Lectotype*** (designated here): Canada, Ontario, Belleville, on cedar (*Thuja occidentalis*) stump in a swamp, 5 miles west of Belleville, Ont. *J*. *Macoun* & *N*. *C*. *Kindberg* (PC0132686!).

*Plagiothecium curvifolium* fo. *julaceum* Clum. & E. Bauer, *Musci Europ*. *Exs*. 27: *1307*. 1915 (C-M-9129!, MO3974490!), syn. nov.

***Lectotype*** (designated here): *Musci eur*. *exs*. *1307*, leg. P. Culman, auf Tannenwurzeln ini der Nähe der oberen Waldgrenze, Burgfeld ob Beatenberg, Kanton Bern, Switzerland, 1630–1700 m, 31 July 1912 (C-M-9129!, isolectotype: MO3974490!).

**Fig 12:**
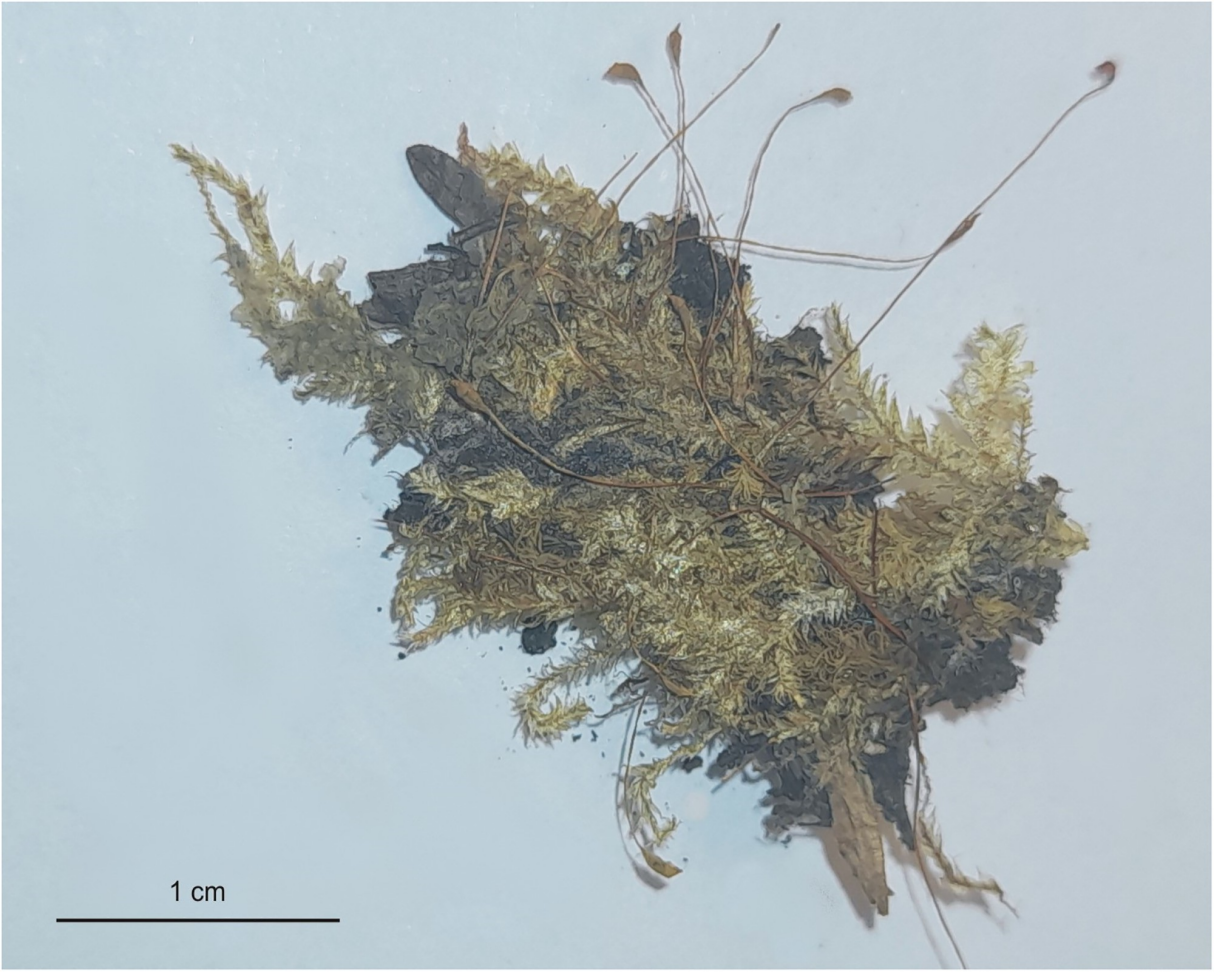
The turf of *Plagiothecium decursivifolium* (from lectotype, PC0132686), photo. G. J. Wolski, 19 November 2021.

**Fig 13:**
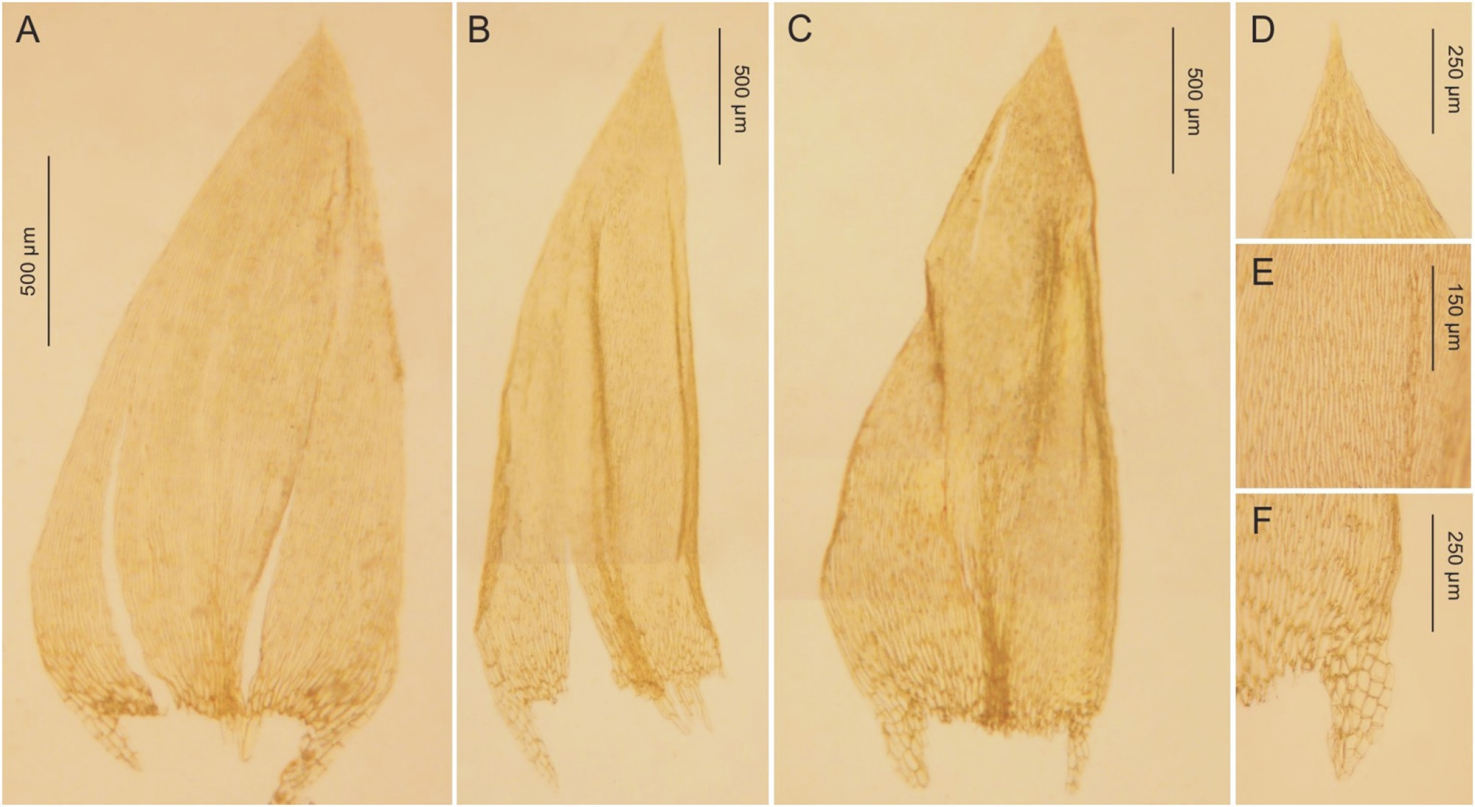
The most important taxonomic features of *Plagiothecium decursivifolium*. A–C—stem leaves (A—*J*. *T*. *Wynns 1939*, CP0010621, B–C—from lectotype, *P*. *Culmann*, C-M-9120); D—leaf apex; E—cells from the middle part of the leaf; F—decurrencies (D–F from lectotype *P*. *Culmann*, C-M-9120), photo. G. J. Wolski, 19 November 2021.

**Fig 14:**
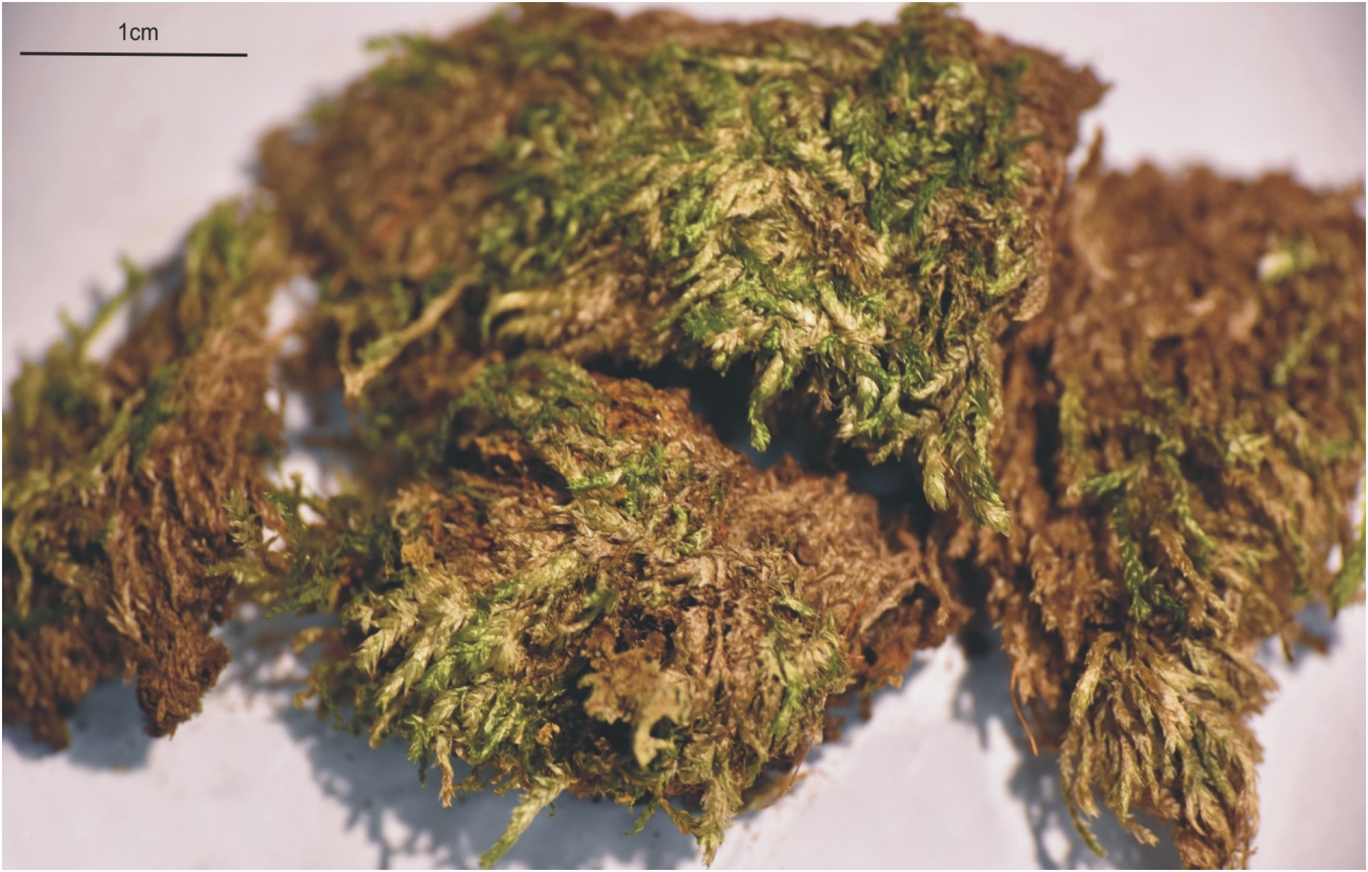
The turf of *Plagiothecium imbricatum* (from holotype *Wolski 424*, LOD 15015), photo. G. J. Wolski, 14 September 2021.

***Description***: Plants medium-sized to small, yellow to yellow-green, with a metallic luster (Fig 12); stems gently julaceous and imbricate, 0.6–1.5 cm long; leaves folded, ovate, ovate-lanceolate, concave, therefore often cracked at the base, slightly curved towards the ground. Leaves asymmetrical, those from the middle of the stem 1.3–2.5 (M 1.8) mm long, and the width measured at the widest point 0.4–1.8 (M 0.9) mm; margin sometimes slightly incurved on both sides; base wide; the apex acuminate, not denticulate or rarely with one tooth; costae two, variable, but quite thick, extending even to ½ leaf length, reaching 0.1–1.4 (M 0.5) mm; cells from the midleaf linear-vermicular, 95–190 (M 150) × 6–10 (M 8) μm, areolation tight; decurrencies 270–360 (M 315) × 80–125 (M 102) μm, forming distinct auricles (Fig 13), created by 3–5 rows of rectangular, quadrate, quite often inflated cells (Fig 14), 60–70 × 26–30 μm; sporophytes 1.0–1.3 cm long; capsules inclined, cylindrical, 1.5–1.6 × 0.5–0.6 cm; operculum rostrate.

***Distribution and ecology***: the presently known range of *P*. *decursivifolium* are Europe, Asia and North America. In this area, it is mainly recorded from mixed and coniferous forests (dominated by *Picea*), less often from *Pinus* (including also *Pinus* monocultures), *Abies*, *Fagus* or *Alnus* dominated forests. This species most often was found in epigeic habitats (on humus, mineral soil), less often was it epiphytic (*Betula pendula*, *Quercus* sp., *Pinus sylvestris*, *Picea abietis*, *Abies alba*), epixylic (trunk, log), or epilithic (rock) or in anthropogenic habitats such as old railroads (S1 Text). ***Additional specimens examined*:** as a supplementary materials (S1 Text).

***Plagiothecium imbricatum*** G.J.Wolski & W.R.Buck, sp. nov.

***Type***: Poland, kujawsko-pomorskie Voivodeship, surroundings of Dolina rzeki Brdy reserve, slope near the river on soil in mixed forest, 13 July 2020, *G*. *J*. *Wolski*, *Wolski 424* (holotype: LOD 15015!, isotypes: NY04688394!, SZUB-B 00001!).

**Fig 15:**
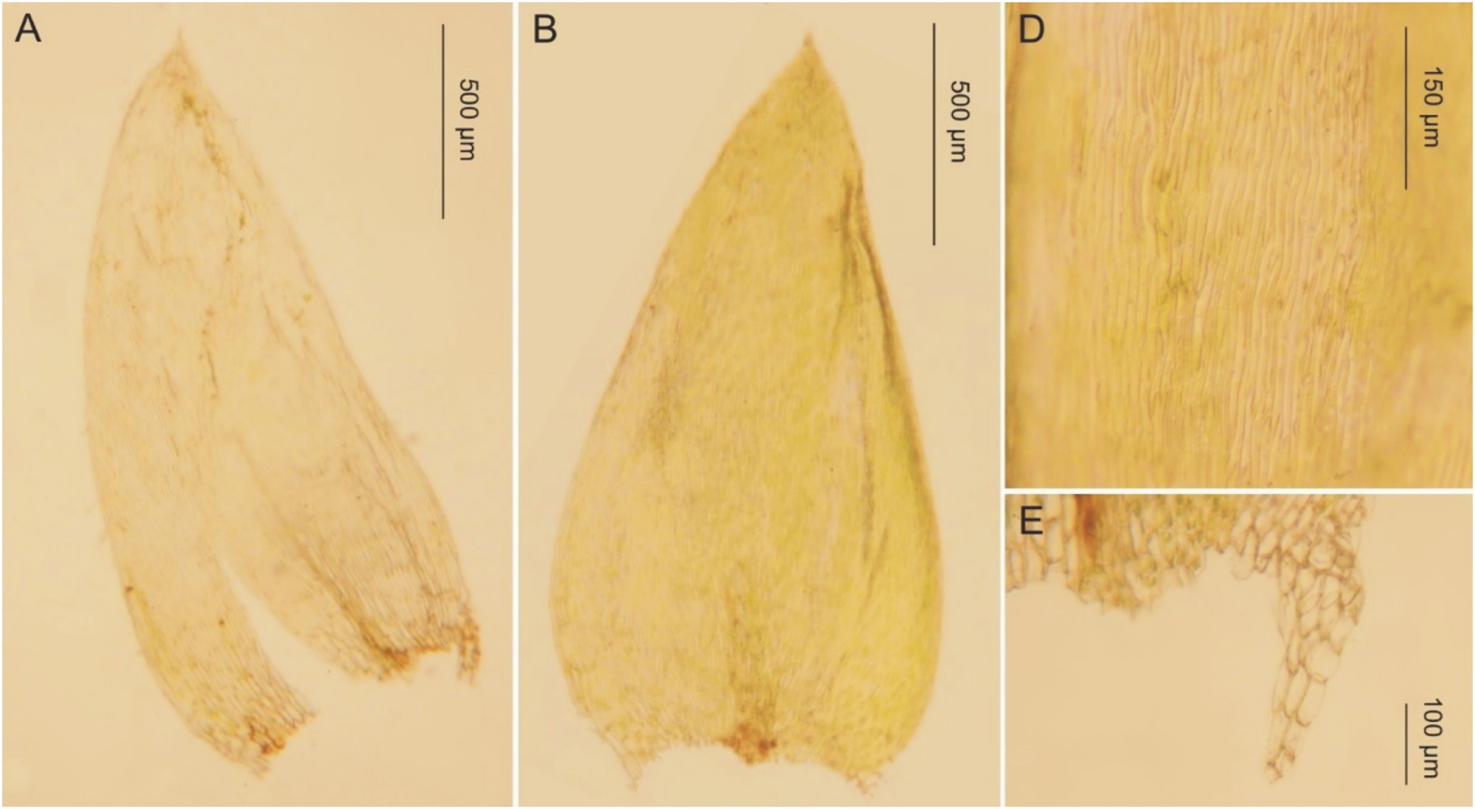
The most important taxonomic features of *Plagiothecium imbricatum*. A–C—asymmetric stem leaves; D—cells from the middle part of the asymmetric leaf; E—decurrencies of asymmetric leaves; F–H—symmetric stem leaves; I—cells from the middle part of the symmetric leaf; J—decurrencies of symmetric leaves (A–J from holotype *Wolski 424*, LOD 15015), photo. G. J. Wolski, 14 September 2021.

***Description***: Plants small, bright-green to green, with a metallic luster (Fig 14); stems clearly julaceous and imbricate, 0.7–1.5 cm long, densely foliate; two types of leaves: symmetrical and asymmetrical. The symmetrical ones folded, lanceolate, concave, sometimes strongly cracked at the base, asymmetrical ones ovate, slightly concave or flat, both types of leaves identical in size, those from the middle of the stem 1.2–2.3 (M 1.7) mm long, and the width measured at the widest point 0.7–1.0 (M 0.8) mm; margin plane; the apex acuminate, not denticulate; costae two, short and thin, extending from 1/5 to 1/3 leaf length, reaching 120–700 (M 400) μm; cells from the midleaf linear-vermicular, 80–190 (M 140) × 5–9 μm, cell areolation very tight; decurrencies 250–270 × 95–100 μm, forming distinct auricles, created by 3–4 rows of rectangular, quadrate, quite often inflated cells (Fig 15), 65–70 × 20–25 μm; sporophytes unknown so far.

***Distribution and ecology***: currently known range of *P*. *imbricatum* is mainly central Europe, single positions are given from northern and western Europe and North America. From this area this species mainly is recorded from mixed and *Fagus* forests, it also is noted mainly in epigeic habitats (on humus and mineral soil, slopes), less often on anthropogenic habitats (such as drainage ditches), and on the bark of living trees (S1 Text).

***Additional specimens examined*:** as a supplementary materials (S1 Text).

### Key to *Plagiothecium curvifolium sensu lato*

**1.** Plants rather medium-sized; foliage complanate, leaves not cracked at the base; decurrencies not forming distinct auricles, inflated cells rather absent … **2.**

**1’.** Plants medium-sized or small; stems more or less julaceous and imbricate or clearly julaceous and imbricate; leaves often or sometimes cracked at the base; decurrencies forming quite distinct auricles, inflated cells often present … **3.**

**2.** Leaves slightly curved towards the ground; symmetrical leaves dominating, 1.7–2.7 (M 2.2) × 0.7–1.5 (M 1.0) mm; apex usually not denticulate; cells from the midleaf 110–151 (M 130) × 8–9 μm; sporophytes short, 1.3–1.6 cm long; operculum conical, obtuse … ***P*. *curvifolium* var. *curvifolium*.**

**2’.** Leaves early curved towards the ground; asymmetrical, hooked leaves dominating, 1.7–2.2 (M 2.0) × 0.6–0.9 (M 0.75) mm; apex usually denticulate by 2–3 teeth; cells from the midleaf 60–120 (M 100) × 7–9 μm; sporophytes 1.7–2.5 cm long; operculum rostellate … ***P*. *curvifolium* var. *recurvum*.**

**3.** Plants not clearly julaceous and imbricate; asymmetrical leaves dominating; cells from the midleaf 95–190 (M 150) × 6–10 (M 7) μm; decurrencies created by 3–5 rows of rectangular, quadrate, quite often inflated cells; operculum rostrate … ***P*. *decursivifolium*.**

**3’.** Plants small, clearly julaceous and imbricate; two types of leaves, symmetrical and asymmetrical; cells from the midleaf 80–190 (M 140) × 5–9 μm; decurrencies created by 3–4 rows of rectangular, quadrate, quite often inflated cells … ***P*. *imbricatum*.**

## Discussion

Many articles indicated that the *P*. *curvifolium sensu lato* is very variable [28,29,37,44–48], while this variability is mainly related to the qualitative and quantitative features of the gametophyte (e.g. the symmetry, dimensions, concavity of leaves; cell length; serration of the leaf apex; the shape of the decurrencies). However, no specific research into the causes of this variability has been undertaken so far [28,29,37,44–48]. Conducted studies on the intraspecific variability of *P*. *curvifolium sensu lato* show that it is a complex comprising four separate taxa: *P*. *curvifolium* var. *curvifolium*, *P*. *curvifolium* var. *recurvum*, *P*. *decursivifolium* and *P*. *imbricatum*.

For decades, the genus *Plagiothecium* has hardly been the subject of any research. It is changing very intensively now, and molecular research, which are now commonly used in taxonomy, along with other methods help not only explain the intraspecific variability of problematic taxa, but also shed new light on the relationship between closely related species [2,3].

During the research all tested specimens, types (*P*. *curvifolium* JE04004091; *P*. *curvifolium* fo. *julaceum* C-M-9120, MO3974490; *P*. *curvifolium* var. *hypnophyllum* VLA; *P*. *denticulatum* var. *recurvum* HBG02115, HBG, JE04004201, PC01322640, WRSL; *P*. *decursivifolium* PC0132686), as well as the protologues of each name [4,9,14,15,18,21,23,24,40,49] are different in qualitative and quantitative characteristics. However, the differences between the individual specimens relate primarily to: plant size; arrangement of leaves on the stem; the symmetry, dimensions, shape, concavity and folding of leaves; cell length; serration of the apex; the shape of the decurrencies; the length of the sporophyte and the shape of the operculum (Figs 10–15).

We agree with what is stated by Wynns [2] that among *P*. *curvifolium sensu lato* other taxa can be distinguished, however for a taxon with a feature *P*. *curvifolium* fo. *julaceum* there is another previously published name representing the same characteristics–*P*. *decursivifolium*.

Our research proposes a resurrection of *P*. *decursivifolium* [58]. In the diagnosis, Macoun & Kindberg [58] stated that it is intermediate between *P*. *latebricola* Wilson *ex* Schimp. and *P*. *pseudo*-*latebricola* Kindb. *in* Macoun. Despite the similarity of the name (*P*. *decursivifolium* and *P*. *curvifolium*) in both cases indicating curved leaves, lack of comparison *P*. *decursivifolium* to *P*. *curvifolium* is related to the fact that it was not distinguished by bryologists at the end of 19^th^ and at the beginning of 20^th^ centuries [e.g., 5–8].

Macoun & Kindberg [58] in the diagnosis wrote that *P*. *decursivifolium* is characterized, e.g., by flattened foliage; inclined capsules; and a curved operculum. Grout [15], who quoted the type of *P*. *decursivifolium*, indicated that it is a form of *P*. *latebricola* „with narrower leaf cells, about 5–6 μ wide”. While Ireland [27] synonymized this taxon with *P*. *laetum*, he wrote „*P*. *laetum* (…) has two forms (…). The most common form has (…) capsules that are smooth, straight and usually erect. Plants of this description have been named *P*. *decursivifolium*”. This point of view was adopted by and is recognized so far [61].

Whereas, Wynns [2] reported that specimens of this taxon erroneously described as "types" are deposited in NY Herbarium (NY164182, NY164138), and he added that these materials represent *P*. *latebricola*. Indeed, these specimens appear to be the mentioned taxa. However, a detailed analysis of the description of the envelopes of these materials (NY164182!, NY164138!, both available online) showed that, habitat „on earth”, location „Ottawa”, as well as the date of collection „Oct. 5, 1907”, as well as features of these specimens are inconsistent with the diagnosis. Therefore these specimens cannot be types of *P*. *decursivifolium*, which confirms the premises given by Wynns [2]. Additionally, in the Natural History Museum, Herbarium BM a specimen (BM13777462) signed as „probable original material *Hypnum* (*Plagiothecium*) *latebricola* Lindb.” has been found. Based on description of habitat, location and a reference to *H*. *Passaicense* Austin, it reflects the data contained in the aforementioned diagnosis quite well. However, the features of this specimen are also inconsistent with the characteristics stated in diagnosis of *P*. *decursivifolium* as in the case of specimens from NY Herbarium.

Detailed diagnosis analysis of all the species mentioned above, in particular diagnosis of *P*. *decursivifolium* and the characteristics given for this taxon [58] exclude not only *P*. *latebricola*, but also *P*. *laetum*, because none of them is characterized by, e.g., inclined capsules and a curved operculum [62]. Taking into account the above facts, above-mentioned specimens cannot be considered as original collection on which this species was described.

Features characterizing the resurrected species, e.g., quite wide cells 6–10 (M 8) μm; decurrencies forming distinct auricles, created by 3–5 rows of rectangular, quadrate, quite often inflated cells could cause many researchers to mention the possibility of confusion of *P*. *curvifolium sensu lato* (including this taxon) with the *P*. *denticulatum* complex [e.g., 29,37]. However, its genetic distinctiveness and unambiguous morphological features support its restoration and treatment as a separate species.

In the 20^th^ and 21^st^ centuries, bryologists not only pointed to the problematic aspect of *P*. *curvifolium sensu lato*, but also reported a large range of many diagnostic features of this taxon. Mentioned, e.g., symmetrical or nearly symmetrical to asymmetrical leaves; margin entire or with denticulations at apex; cells from the middle part of the leaf 80–160 × 6–10 μm [28–29,37,44–47], at present explain the intraspecific variability of the described complex and help to separate individual taxa from each other.

As reported by Wynns et al. [50] in genetic analyzes, *P*. *curvifolium* forms a clade with the „widely distributed austral species *P*. *lucidum*”. Our research shows similar relationships and places *P*. *curvifolium* as more closely related to *P*. *lucidum* (Southern Hemisphere) than to *P*. *laetum* (Northern Hemisphere), which is usually considered as closely related with *P*. *curvifolium*. Obviously, these results confirm the legitimacy of the discussed distinction of *P*. *curvifolium* and *P*. *laetum* as separate [27–28,36–37,39], however, they pose a number of new questions about the relationship between the species of both hemispheres, as *P*. *curvifolium* and *P*. *lucidum* or *P*. *schofieldii* G.J.Wolski & W.R.Buck and *P*. *lamprostachys* (Hampe) A.Jaeger and others.

Despite the fact that over the last decades the genus *Plagiothecium* has not been the subject of detailed studies, recent years indicate that it is changing intensively [2–3,50–52,63,64]. Extensive research focused on the taxonomic revision of many problematic taxa not only allows to describe their intraspecific variability, but also allows the description of new species. *Plagiothecium imbricatum* is another species of this genus described over several years from a well-studied lowland area of central Europe [3,52]. These studies, as well as other reviews published in the past few years [50,51], indicate that the whole genus *Plagiothecium*, as well as many similar plagiotorphic genera, still requires detailed taxonomic studies.

## Conclusion

1. So far, *Plagiothecium curvifolium sensu lato* was considered to be one widespread species.
2. The conducted research shows that the described taxon is a complex consisting of four separate taxa–*P*. *curvifolium* var. *curvifolium*, *P*. *curvifolium* var. *recurvum*, *P*. *decursivifolium* and *P*. *imbricatum*.
3. The most important features distinguishing the studied taxa are related to plant size; arrangement of leaves on the stem; the symmetry, dimensions, shape, concavity and folding of leaves; cell length; serration of the leaf apex; the shape of the decurrencies; the length of the sporophyte and the shape of the operculum.

## Supporting information

S1 Text Selected examined specimens *Plagiothecium curvifolium sensu lato*. (DOCX)

## References

[1] Ros RM, Cano MJ, Guerra J. Bryophyte Checklist of Northern Africa. J. Bryol. 1999;21: 207–244. doi: 10.1179/jbr.1999.21.3.207

[2] Wynns JT. Molecular phylogeny and systematic revision of the pleurocarpous moss genus *Plagiothecium*. Ph. D. Copenhagen. University of Copenhagen; 2015.

[3] Wolski GJ, Nowicka-Krawczyk P, Buck WR. *Plagiothecium schofieldii*, a new species from the Aleutian Islands (Alaska, USA). PhytoKeys. 2021;184: 127–138 doi: 10.3897/phytokeys.184.69970 34785976 PMC8589775

[4] Limpricht KG. Die Laubmoose Deutschlands, Oesterreichs und der Schweiz. E. Kummer, Leipzig; 1897.

[5] Braithwaite R. The British Moss Flora. L. Reeve & Co., London; 1896–1905.

[6] Klinggraeff H. Die Leber- und Laubmoose West- und Ostpreussens. Danzig; 1893.

[7] Héribaud J. Les muscinée d’Auvergene. Mémoires de I’Académie des Sciences, Belle-Lettres st Arts de Clermont-Ferrand; 1899.

[8] Bomansson JO. Ålands mosser. Acta Societatis Pro Fauna et Flora Fennica; 1900.

[9] Meylan C. Catalogue des Mousses du Jura. Bulletin de la Société Vaudoise das Sciences Naturalles. 1905;152: 41–172.

[10] Dixon HN. The Student’s Handbook of British Mosses. Third Edition, Eastbourne, London; 1924.

[11] Podpěra J. Conspectus Muscorum Europaeorum. Nakladatelstvi Československé Akademie Věd, Prague; 1954.

[12] Dixon HN. The Student’s Handbook of British Mosses. V. V. Sumfield, Eastbourne; 1896.

[13] Dixon HN. The Student’s Handbook of British Mosses. V. V. Sumfield, Eastbourne; 1904.

[14] Spruce R. Musci praeteriti. Journal of Botany, British and Foreign. 1880;18: 289–295, 353–362.

[15] Grout AJ. Moss Flora of North America North of Mexico. Published by the author, Newfane; 1932.

[16] Paris EG. Index Bryologicus. Paris; 1905.

[17] Loeske L. Beiträge zur Moosflora des Harzes. Verhandlungen des Botanischen Vereins für die Provinz Brandenburg und die Angrenzenden Länder. 1903;43: 80–100.

[18] Warnstorf C. Kryptogamenflora der Mark Brandenburg und angrenzender Gebiete Zweiter Band. Laubmoose. Gebrüder Borntraeger, Leipzig; 1906.

[19] Brotherus VF. Musci (Laubmoose). III Unterklasse Bryales: II. Gruppe: Pleurocarpi. In: Engler A, Prantl K editors. Die natürlichen Pflanzenfamilien, Tiel 1, Abt. 3, Hälfte 2. W. Engelmann, Leipzig; 1909. Pp. 701–1246.

[20] Brotherus VF. Enumeration Muscorum Caucasi. Acta Societatis Scientiarum Fennicae; 1923.

[21] Mönkemeyer W. Die Laubmoose Europas: Andreaeales–Bryales. In: Rabenhorsts L, editors. Kryptogamen-Flora von Deutschland, Österreich und der Schweiz. Vierter Band, Ergänzungsband. Akademischen Verlagsgesellschaft, Leipzig; 1927. pp. 1–960.

[22] Jansen C. Skandinaviens Bladmoosflora. E. Munksgaard, Copenhagen; 1939.

[23] Jedlička J. Monographia specierum europeaerum gen. *Plagiothecium s*. *s*. Spisy Vydávané Přìrodovědeckou Fakultou Masarykovy University; 1948.

[24] Jedlička J. Monographia specierum europeaerum gen. *Plagiothecium s*. *s*. Icones. Spisy Vydávané Přìrodovědeckou Fakultou Masarykovy University; 1950.

[25] Kindberg NC. Additions to the North American and European Bryology (Moss-Flora). Reprinted from The Ottawa Naturalist. Vol. XIV, 1900;5: 78–88.

[26] Cardot J, Thériot I. New or unrecorded mosses of North America. II. Botanical Gazette. 1902;37: 363–380.

[27] Ireland RR. A taxonomic revision of the genus *Plagiothecium* for North America, north of Mexico. National Museum of Natural Sciences Publications in Botany, The National Museums of Canada, Ottawa, Canada. 1969;1: 1–18.

[28] Iwatsuki Z. A revision of *Plagiothecium* and its related genera from Japan and her adjecent areas, I. J. Hattori Bot. Lab. 1970;33: 331–380.

[29] Lewinsky J. The family Plagiotheciaceae in Denmark. Linbdergia. 1974;2: 185–217. https://www.jstor.org/stable/20149227.

[30] Ireland RR. The genus *Plagiothecium* in North America. Evansia. 1985;2: 4–9.

[31] Ireland RR. Synpsis of the genus *Plagiothecium* in North America. Lindbergia. 1986;12: 49–56.

[32] Crum H, Steere WC, Anderson LE. A new list of mosses of North America north of Mexico. The Bryologist. 1973;76: 85–130.

[33] Anderson LE, Crum HA, Buck WR. List of the mosses of North America north of Mexico. The Bryologist. 1990;93: 448–499. doi: 10.2307/3243611

[34] Wolski GJ. Reassessing the taxonomic diversity of *Plagiothecium* section *Orthophyllum* in the North American bryoflora. Brittonia. 2020;72: 337–350. doi: 10.1007/s12228-020-09631-y

[35] Sakurai K. Classification of the genus *Plagiothecium* in East Asia. Botanical Magazine (Tokyo). 1949;62: 111–120.

[36] Iwatsuki Z. Catalog of the Mosses of Japan. Hattori Bot. Lab. 1991; 1–182.

[37] Noguchi A. Illustrated Moss Flora of Japan, Part 5. Hattori Bot. Lab. Nichinan. 1994; pp. 1013–1253.

[38] Iwatsuki Z. New Catalog of the Mosses of Japan. J. Hattori Bot. Lab., Nichinan, 2004;96: 1–182. doi: 10.18968/jhbl.96.0_1

[39] Suzuki TA. Revised new catalog of the mosses of Japan. Hattoria. 2016;7: 9–223. doi: 10.18968/hattoria.7.0_9

[40] Ukrainskaya GJ. De taxis interaspecifis generis *Plagiothecium* Schimp. *in* B.S.G. notula. Novosti Sistematiki Nizshikh Rastenii. 1996;31: 179–185.

[41] Ignatov MS, Afonina OM, Ignatova EA, Abolina A, Akatova TV, Baisheva EZ, et al. Check-List of mosses of East Europe and North Asia. Arctoa. 2006;15: 1–130.

[42] Hodgetts NG, Söderström L, Blockeel TL, Caspari S, Ignatov MS, Konstanova NA, et al. An annotated checklist of bryophytes of Europe, Macaronesia and Cyprus. J. Bryol. 2020; 42: 1–116. doi: 10.1080/03736687.2019.1694329

[43] Blockeel TL, Bell NE, Hill MO, Hodgetts NG, Long DG, Pilkington SL, et al. A new checklist of the bryophytes of Britain and Ireland. J. Bryol. 2020;43: 1–51, doi: 10.1080/03736687.2020.1860866

[44] Li D-K, Ireland RR. Plagiotheciaceae. In: Hu R-L, Wang Y-F, Crosby MR editors. Moss Flora of China. English Version, vol. 7, Science Press, Beijing and Missouri Botanical Garden Press, St. Louis; 2001. pp. 219–242.

[45] Smith AJE. The Moss Flora of Britain & Ireland. Cambridge University Press, Cambridge; 2001.

[46] Nyholm E. Moss Flora of Fennoscandia. II, Musci. Fasciele 5. Lund. The Botanical Society of Lund.; 1965.

[47] Cano MJ. *Plagiothecium* Schimp. In: Guerra JJ, Cano MJ, Brugués M editors. Flora Briofítica Ibérica. Bryophytes of the Iberian Peninsula; 2018. pp. 276–294.

[48] Barkman JJ. Het geslacht *Plagiothecium* in Nederland. Buxbaumia. 1957;11: 13–29.

[49] Geheeb A. Bryologische Notizen aus dem Rhöngebirge. VII (Schluss). Allgemeine Botanische Zeitschrift. 1909;15: 186–192.

[50] Wynns JT, Munk KR, Lange CBA. Molecular phylogeny of *Plagiothecium* and similar hypnalean mosses with a revised sectional classification of *Plagiothecium*. Cladistics. 2017;37: 469–501. doi: 10.1111/cla.12210 34649367

[51] Ignatova EA, Fedorova AV, Kuznetsova OI, Ignatov MS. Taxonomy of the *Plagiothecium laetum* complex (Plagiotheciaceae, Bryophyta) in Russia. Arctoa. 2019;28: 28–45.

[52] Wolski GJ, Nowicka-Krawczyk P. Resurrection of the *Plagiothecium longisetum* Lindb. and proposal of the new species–*P*. *angusticellum*. 2020; PLoS ONE 15(3): e0230237. doi: 10.1371/journal.pone.0230237 32160254 PMC7065767

[53] Katoh K, Rozewicki J, Yamada KD. MAFFT online service: multiple sequence alignment. interactive sequence choice and visualization. Brief. Bioinform. 2017; 1–7. doi: 10.1093/bib/bbx108 28968734 PMC6781576

[54] Lanfear R, Frandsen PB, Wright AM, Senfeld T, Calcott B. PartitionFinder 2: new methods for selecting partitioned models of evolution for molecular and morphological phylogenetic analyses. Molecular Biology and Evolution. 2016; doi: 10.1093/molbev/msw260 28013191

[55] Trifinopoulos J, Nguyen LT, Von Haeseler A, Minh BQ. W-IQ-TREE: a fast online phylogenetic tool for maximum likelihood analysis. Nucleic Acids Res. 2016; 44(W1): W232–W235. doi: 10.1093/nar/gkw256 27084950 PMC4987875

[56] Hoang DT, Chernomor O, Von Haeseler A, Minh BQ, Vinh LS. UFBoot2: improving the ultrafast bootstrap approximation. Molecular Biology and Evolution. 2018; 518–522. doi: 10.1093/molbev/msx281 29077904 PMC5850222

[57] Ronquist F, Teslenko M, Mark van der P, Ayres DL, Darling A, Höhna S, et al. MrBayes 3.2: efficient Bayesian phylogenetic inference and model choice across a large model space. Syst. Biol. 2012;61: 539–542. doi: 10.1093/sysbio/sys029 22357727 PMC3329765

[58] Macoun J, Kindberg NC. Catalogue of Canadian Plants, Part IV.–Musci. Geological and Natural History Survey of Canada, Ottawa. 1892; 1–295.

[59] Turland NJ, Wiersema J, Barrie FR, Greuter W, Hawksworth DL, Herenden PS, et al. International Code of Nomenclature for algae, fungi, and plants (*Shenzhen Code*) adopted by the Nineteenth International Botanical Congress Shenzhen, China, July 2017. Regnum Vegetabile 159. Koeltz Botanical Books, Glashütten; 2018. 10.12705/Code.2018.

[60] Warnstorf C. Moosflora der Provinz Branderburg und die Angrenzenden Länder; 1885.

[61] Flora of North America, www.eFloras.org, accessed 22 December 2021. http://www.efloras.org/florataxon.aspx?flora_id=1&taxon_id=10700.

[62] Bruch P, Schimper WP, Gümbel WT. Bryologia Europaea Seu Genera Mscrum Europaeorum Monographice Illustrata. In: Schimper WP edsitors. Sumptibus Librariae E. Schweizerbart, Stuttgart, Germany, vol. 5; 1851.

[63] Wolski GJ, Nowicka-Krawczyk P, Buck WR. *Plagiothecium talbotii*, a new species from the Aleutian Islands (Alaska, U.S.A.). PhytoKeys. 2022:194 67–73. doi: 10.3897/phytokeys.194.81652 35586326 PMC9033742

[64] Wolski GJ, Nowicka-Krawczyk P, Buck WR. *Plagiothecium schofieldii*, a new species from the Aleutian Islands (Alaska, USA). PhytoKeys. 2022:184 127–138. doi: 10.3897/phytokeys.184.69970 34785976 PMC8589775

