# Supplementary material for: Taxonomic revision of the Plagiothecium curvifolium complex
Source: PLoS One. 2022 Nov 9;17(11):e0275665. doi: 10.1371/journal.pone.0275665 (PMC9645594; doi:10.1371/journal.pone.0275665)
Supplement: S1 Text — (DOCX) [file pone.0275665.s001.docx]

Selected examined specimens *Plagiothecium curvifolium sensu lato*

***Plagiothecium curvifolium* var. *curvifolium* Schlieph. *ex* Limpr.**

**Belgium**: Prov. de Liége., Ternell (Eupen), sur le sol, au pier d’un heter, a proxim, de la Helle, un peu en aval du confluent du Ternellbach, 13 Nov. 1960, *J*. *Lambinon 60/B/2390*, H3112257. **Canada**: British Columbia, road to Menhininck Rd., Saltspring I, rotten wood, 09 May 1962, *F*. *Bas 343a*, C-M-34500; Nova Scotia, Kejimkujik Natl. Park, coniferous closed crown forest, 300 old hemlock stand along N shore of Big Dam Lake, tree base, 44°27’N, 65°15’W, 11 Jun. 1992, *R*.*J*. *Belland*, *W*.*B*. *Schofield 16791*, ACC B146596, trial from Jake’s Landing toward Merrymakedge, Kejimkujik Lake, on rock, 44°23’N, 65°13’W, 14 Jun. 1992, *W*.*B*. *Schofield 97257*, *R*.*J*. *Belland*, ACC B154070. **Czech Republic**: Waldboden am Wege beim, J.H. Joachimsthal, 900 m, 21 Aug. 1898, *E*. *Bauer*, YU173297, BM14148091. **Denmark**: Bornholm, Svartingedalen, v. Svartingegård, 31 Dec. 1972, *J*. *Lewinsky*, C-M-34610, Hammeren, SV naer det gamle fyr, 31 Dec. 1972, *J*. *Lewinsky*, C-M-34609, 02 Nov. 1972, C-M34592; Gribskov kommune, disturbed woods by Storkevad Trinbraet, a whistle stop, 1.3 km W of parking area at jct Storkevadsvej & Gillelejevej (Hwy. 227), Gribskov, Sjaelland, on banks of Smap creek just E of track, 56°01’12’’N, 12°19’48’’E, 31 Aug. 2010, *J*.*T*. *Wynns 2149*, C-M-10520; Jylland, Silkeborg v. Ørnsø, 22 May 1968, *K*. *Holmen*, C-M-34689; Rudersal kommune, around Agersø, E of Hørsholm Kongevej (Hwy. 229), Rude Skov. Sjaelland, on humusy root-ball of fallen birch at lake, 55°50.6’N, 12°28.9’E, 09 Apr. 2012, *J*.*T*. *Wynns 2038*, *N*. *Jacobsen*, C-M-10516; Sjaelland, Horbaek Plantage, Jul. 1881, *C*. *Jansen*, H3112229. **Estonia**: Ad basin arborum in silva Rouanurme prope Wastselina, dist. Worumaa, paroch. Wasrselina, 30 Jul. 1919, *D*.*W*.*J*. *Reinthal*, *N*. *Malta*, H3112269. **Finland**: Kuhmoinen, Tehinniemi Leiniemi, Sandgrube, 90 m, 61°34’N, 25°11’E, 22 Jul. 1971, *Eberhard*, *P*. *Hegewald 3727*, MO5240616. **France**: Haute-Loire, Saint-Juliec-Molhesabate, pentes nord de la cote, 1359, prés du Felletin, bois résineux, sur l’humus, 1200 m, *P*. *Cuynet 227*, PC0784976; Loire, Massif du Pilat, sous-bois résineux des pentes nord-est, a terre, 1300 m, 17 Oct. 1954, *P*. *Cuynet 678*, PC0784971, pentes nort-est, au pied d’un spin, sur l’humus (station séche), 1200 m, 30 Aug. 1953, *P*. *Cuynet 435*, PC0784973; Puy-de-Dome, Chambon-sur-Lac, pentes boisées au S.O. du Lac, 16 Aug. 1961, *P*.*B*. *Pierrot 23969*, PC0784958. **Georgia**: Grosser Kaukasus, Zentraltei, Chewsuretien, Ttogtal, 500 m, nordöstlich Khone, an Totholz unter lichten Birkenwäldern, 2200 m, 42°34’38’’N, 45°14’39’’W, 02 Sep. 2006, *H*.-*J*. *Zündorf 23354*, *R*. *Marstaller*, MO6627130. **Germany**: Hessen, Bidenkopf in *Piceeto abietis*, 600m, 29 Dec. 1955, *H*. *Roivainer*, H3112299; Thuringia, 1872, *H*. *Bernet 47*, C-M-34505. **Great Britain**: England, Mill wood below Rosebery Reservoir, base of *Quercus* in *Quercus* dominated wood land, 212 m, 5 Feb. 1978, *D*.*G*. *Long* 6489, ACC B242235. **Hungary**: Budapest, ad pedem *Quercus* in sylvestribus montis Nagyharshegy, 10 Dec. 1954, *L*. *Vajda*, H3112278; Fejer, in argilopsis silvat, vallis Csatorna-völgy prop Csakbereny, 250–300 m, 17 Apr. 1949, *A*. *Boros* 18645, PC0784960; Pest, ad pedem Fagi supra vill., Keserüsvölgy prope pag., Visegrad, montes Pilis, 23 Sep. 1956, *L*. *Vajda*, H3112280, in silva mtis Nagyszénás pr. Pag. Nagykovácsi, 17 Feb. 1975, *L*. *Vajda*, in rupestribus villis Mlynica, montes Visoky Tatry, 1100 m, 08 Jul. 1962, *L*. *Vajda*, C-M-34502. **Latvia**: Livland, Kreis Riga, an den Rändern alter Sandgruben im Kiefernwalde S von Kleistenhof, 28 Nov. 1908, *J*. *Mikutowicz* H3112243, C-M-34527, am Grunde einer Brike im Graben des Weges von Grawenheide nach Potenfeld, River Dreilingsbusch, 24 Aug. 1908, *J*. *Mikutowicz*, C-M-34524, am Grunde von Kiefern und Fichten im sog. „Urwald” and as W von Bilderlingshofm 29 Apr. 1907, *J*. *Mikutowicz*, C-M-34526,. **Netherlands**: Hoge Veluwe, N. of Arnhem, 10 May 1969, *K*. *Holmen*, *J*. *Lewinsky*, C-M-34522, C-M-34537. **Poland**: Pomorze Zachodnie, Bory Tucholskie National Park, by the southeastern end of Jezioro Płęsno, 16 Jul. 1998, *S*. *Lisowski B-12910*, MO5648292, Wyspa Wolin, Jezioro Gardno near Grodno I, in beech forest, 08 Aug. 1995, *S*. *Lisowski B-11364*, MO4434941; West Carpathians, Bieszczady Mts., by the ascent from the hotel Muszne to the ridge Bukowe Berdo, 820 m, rather abundant on a patchily rotting tree trunk, 27 Sep. 1999, *C*.*C*. *Townsend 99/51*, MO5628607, Polish Western Beskids, Polica Range, below the summit of Mt Złota Grapa, 1250 m, on the Carpathian spruce forest, 12 Oct. 1987, *R*. *Ochyra*, MO3078123, on northern slope of Mt Złota Grapa, 1100 m, in loose spruce forest, 12 Oct. 1987, *R*. *Ochyra*, MO3078200, Tatra Mts., Podhale Region, Rów Podtatrzański, between Pardołówka and Jaszczurówka in Zakopane, 880 m, on ground in *Picea abies* forest, 16 Jun. 1985, *J*. *Wójcicki*, *R*. *Ochyra*, MO3682584; Western Carpatians, Babia Góra National Park, Krowiarki Pass, spruce forest, 5 Aug. 1997, *K*. *Grodzińska*, *B*. *Godzik*, *R*. *Ochyra*, MO *5135836*, Rów Podtatrzański, Głodówka, 1080 m, in forest dominated with Norway spruce, 42°17’42’’N, 20°06’55’’E, Aug. 2005, *B*. *Godzik*, *B*. *Cykowska*, MO *3370589*. **Romania**: Sinaia, Monte Bucegi, 1290 m, 49°19’N, 25°29’W, tocón de *Picea*, 09 Aug. 2009, *J*. *Guerra*, *M*.*J*. *Cano*, *J*.*A*. *Jiménez* &*J*.*D*. *Orgaz*, MO6766171. **Russia**: Sakhalin Island, Smirnykhovskyi Region, next to the Orlovka River along the road to Palevo village, abt. 25 km from Smirnych village, in the 2^nd^ growth spruce and fir forest across the road from the campsite, 49°43’47”N, 124°31’28”E, 850 ft., on decayed wood in the birch swamp area, in part shade, 26 Jul. 2003, *J*.*A*. *Harpel* 32388, *V*. *Cherdensteva*, ACC B237211. **Spain**: Burgos, Quintanar de la Sierra, Lagunas se Neila, laguna de La Cascada, 42.0501N, 3.0521W, 1704 m, taludes al. borde de la laguna, en bosque de *Pinus sylvestris*, 19 Feb. 2000, *J*. *Muñoz*, MO6623963. **Sweden**: Halland, Enslöv, 1920, *A*. *Hülphers*, H3113444; Lycksele, Lappmark, Tärna, 20 Aug. 1934, *A*. *Hülphers*, H3113447; Södermanland, par. Helgona, Broby, in abiegno, 28 Jul. 1915, *W*. *Arnell*, H3112228; Skåne, Höör sn., south-west side of Dagstorps-sjön, on root of *Alnus*, 25 Jul. 1967, *E*. *Nyholm*, PC0784988; Uppland, Norra Sticklings, Lidingo, large mats on banks, 22 Oct. 1957, *W*.*A*. *Weber*, MO5284493; Västergötl, Borgunda sn. Apr. 1921, *A*. *Hülphers*, H3112217; Västergötland, Nåleskov ved Mellomsjömyren, 31 Jul. 1974, *K*. *Holmen*, *J*. *Lewinsky*, C-M-34565. **U.S.A.**: Michigan, Emmet and Cheboygan Counties, Burt and Douglas Lakes, Jul.–Aug. 1922, *G*.*E*. *Nichols*, YU233887; New Hampshire, Hillsboro Co., on living wood, usually the base of a decic. tree near water, 1961, *H. v. Schneider, E. L. Anderson, A. M. Reid*, herbarium; Pennsylvania, Huntington Co., east and west face of Tussey Mountain, along Pennsylvania Furnace Road, 2000 ft., on sandstone boulders, W side of montain, 21 Apr. 1986, *B*. *Allen* 4880, ACC B101907, Centre Co., Bear Meadows Nat. Moument, 6.5 mi S of State College, around base of various tres, hemlock-hardwood forest with *Rhododendron*, 28 Jul. 1973, *R*.*A*. *Pursell*, *M*.*G*. *Manuel 9999*, ACC B236344; Washington, trail toward Lake Constance, Olympic National Park, humus by trail, 05 Oct. 1965, *W*.*B*. *Schofield 28392*, *Z*. *Iwatsuki*, ACC B24229.

***Plagiothecium curvifolium* var. *recurvum* (Warnst.) G.J. Wolski & W.R. Buck**

**Austria**: Steiermark, NW Rüchen des Stuhlecks, 1500 m, auf Humöschung, 05 Jun. 1960, *J*. *Froehlich*, H3112235. **Belgium**: Prov. de Liège, partic super, de la villée du Getzbach, en amont du Pont Brûlé (Hautes Fagnes), base troc de *Quercus*, bois clair à proximité du ruisseau, 15 Nov. 1960, *J*. *Lambinon 60/B/2434*, H3112258, Waisomont (Ferrières, à la limite comm., de Werbomont), pessière humide à proximité de la grand-route de Bastogen, 08 Oct. 1960, *J*. *Lambinon 60/B/2181*, H3112256. **Canada**: Britsh Columbia, 3 m S of Brandywine Falls, 50°00’N, 123°07’W, *Pinus*, *Pseudotsuga* forest over shallow soil with pond depression and petland, base of tree in forest, 19 Jun 2003, *W*.*B*. *Schofield 121124*, *D*. *Jamieson*, ACC B192553, Bounty Co., ancient *Thuja plicata* grove, rotten log, Jackson Creek trail #311, 48°51’N, 117°00’W, 28 Sep. 2001, *W*.*B*. *Schofield 119044*, ACC B189663; Québec, Forillon Natl. Park, along trail to summit of Mt. St-Alban, 48°48’N, 64°13’W, on shaded mineral soil, 13 Jul. 1989, *R*.*J*. *Belland*, *W*.*B*. *Schofield 13298*, ACC B152158. **Czech Republic**: Bohemia, ad corticem *Piceae* prope opp., Hostinné, 400 m, Jun. 1949, *Z*. *Pilous*, H3112272, C-M-34541; ČSSR, blansko, Moravia Karst, in locis saxosis in Suchý zleb, Aug. 1970, *F*. *Grüll*, H3112294. **Denmark**: Carsten Langes Stifelse, Gammel Køgegards Skov, Sjaelland, 05 Dec. 1972, *K*. *Holmen*, *J*. *Lewinsky*, H3112230; Craved Skov., 12 May 1974, *J*. *Lewinsky*, C-M-34675; Jylland, distr. 14, Mønsted plantagwe, 31 Oct. 1975, *G*.*S*. *Mogensen*, C-M-34577; På jord, Draved Skov, Lindestykket, 20 Dec. 1975, *J*. *Lewinsky*, *L*. *Rasmussen*, C-M-34579; Stevns kommune, Gjorslev Bøgeskov, a coastal forest at the S end of Køge Bugt, Sjaelland, on soil (forest stump) in deciduous wood, 55°22’N, 12°24’E, 28 Mar. 2010, *J*.*T*. *Wynns*, C-M-10518; St. Arden Skov, cross roads 1 km west of lake Madum, Jylland, 19 Sep. 1970, *J*. *Lewinsky*, H3112231, Vejkryde 1 km, west from Madum Sø., 19 Dec. 1969, *J*. *Lewinsky*, C-M034676. **Finland**: Alandia, Jomala, Jättböle, ad radices *Piceae abietis*, 25 May 1961, *X*. *Laila*, *H*. *Roivainen*, PC0784987; Kb. Juuka, Polela, Takkovaara dolomite region between the lakes Hanhilampi and Valkealampi, 05 Aug. 1972, *J*. *Lewinsky*, C-M-34553; Pohjois-Pohjanmaa, Muhos, Leppiniemi, S. shore 0.5 km downstream of Pyhäkoski hydro-electric power station, middle slope, 35 m, damp spruce-dominated forest on steep stream side bank, on turf by fallen tree, 29 Dec. 1974, *M*. *Ohenja*, *T*. *Ulvinen*, C-M-34555; Sb. Kaavi, Nunivaara, the serpentune rocks at the E-shore of Lake Louhilampi, 05 Aug. 1972, *J*. *Lewinsky*, C-M-34566. **France**: Beaurepaire, vallon, talus forestier, 23 Dec. 2003, *J*.*Werner 7437*, PC0790451; Languedoc-Roussillon, Lozére, peuplement de Douglas, 1185–1245 m, *J*. *Baedat*, PC0762052; Loire, co. de la Croix de l’Homme mort, bois de résineux, 12 Aug. 1961, *P*.*B*. *Pierrot 23969*, PC0784958; Loire-Monts du Forez, mont de Courreau, bois de Regardiére, souche porrissante, 1100 m, 27 Jun. 1954, *P*. *Cuynet*, PC0784975, Saint-Julin-Molhesabate, pentes nord de la cote 1359, prés du Fellatin, bois résineux, sur l’humus, 1200 m, 28 Jun. 1953, *P*. *Cuynet 19966*, PC07844964, pentes boisées de la cote, 1369 m, a terre, 1200 m, *P*. *Cuynet* *19829*, PC0784956; Seine, bois de Verriéres sur souche, 03 Mar. 1912, *F*. *Camus*, PC0022804. **Germany**: Achtermann, Harz, Stein, 07 Nov. 1965, *E*. *Ererhard*, *P*. *Hegewald 500*, MO5285677; Berlin-Grunewald, Jagen 37, Waldboden (Kiefer), 23 Feb. 1966, *E*. *Hegewald*, *P*. *Hegewald 405*, MO5285547; Ebbegebire, Fürwigge-Strasse, 51°08’N, 07°46’E, *E*. *Hegewald*, *P*. *Hegewald 2328*, MO5240613; Hessen, Landkr., Fulda, Rhön-Gebirge, Kaskadenschlucht des Feldbaches, nordöstl., von Sandberg b. Gersfeld, 22 Sep. 1975, *E*. & *P*. *Hegewald*, MO5222636, Laubach, unter Fichten auf dem Gaulskopfe, 300 m, Nov. 1900, *G*. *Roth*, BM14148092; Iserholm, Elsebachtal bei Schwerte (Villigest), 140–190 m, 51°27’N, 07°34’E, *E*. *Hegewald*, *P*. *Hegewald 4108*, MO5240607; Lower Saxony, in trockenen Kiefernwaldern auf humosem Sandboden, in der Umgebung zwischen Kirchorf u. Kuppendorf auf MB, Mendorf, 09 May 1977, *A*. *v*. *Hubschmann 333*, MO5912440; N Heudorf ner Mengen, spruce plantation, 1994, *G*. *Philippi*, H3112295; North Rhine-Westphalia, Somerland, Land Kreis Altena, Teutmecke and Tauberg bei Ohle, 330 m, 51°14’N, 07°49’E, *E*. *Hegewald*, *P*. *Hegewald 3006*, MO5240611; Oberbergischer, Genkeltalsperre, 290 m, 51°03’N, 07°37’E, 18 Dec. 1971, *E*. *Hegewald*, *P*. *Hegewald 4088a*, MO5240614; Wallace *s*.*n*. 25 May 1966, MO5222764. **Great Britain**: England, Hampshire Co., stump in wood, 31 Mar. 1967, *A*.*C*. *Crundwell*, ACC B242234. **Hungary**: Budapest, in sylvestribus vallis Irhásárok, 04 Aug. 1957, *L*. *Vajda*, C-M-34523; Pest, in rupibus umbrosis vallis Holdvilágárok prope pag., Pomáz, 02 Dec. 1960, *L*. *Vajda*, H3112282, in silvestrisibus ad fontem Szentkut in monte Hosszuhegy, prope pag., Csobánka, 02 Apr. 1967, *L*. *Vajda*, H3112283. **Latvia**: Livland, Kreis Riga, am Grunde einer Birke im Graben des Weges, von. Grawenheide nach Rotenfeld, Revier Dreilingsbusch, 24 Nov. 1908, *J*. *Mikutowicz*, H3112237; unter alten Kiefern an Sand-Abhängen, im Kiefernwalde, ½ km SO von Gross-Schmerl an der St.- Petersburger Chaussée, 03 Aug. 1909, *J*. *Mikutowicz*, C-M-34525.**Poland**: Masurian Lakeland, Olsztyn Lake District, 2 km SE of Straszny Dwór in Olsztyn, 130 m, on ground in shaded *Picea abies* forest, 15 Sep. 1985, *L*. *Olesiński*, *R*. *Ochyra*, MO3680606; Mt. Szyndzielnia, 1050 m, on litter in Carpathian spruce forest *Piceetum tatricum*, 17 Sep. 1991, *J*. *Żarnowiec*, *A*. *Stebel*, *H*. *Klama M-438*, MO5127593; Pomorze Zachodnie, Wyspa wolin, near Wisełka, by the road towards the Baltic Sea, in medium-moist pine forest, 09 Sep. 1995, *S*. *Lisowski B-11453*, MO5147825; Rybnik Plataeu, Sumina, on grand in *Pinus sylvestris* forest *Leucobryum-Pinetum*, 29 Jul. 1993, *A*. *Stebel M-255*, MO5127592; Silesian Beskid, Istebna prov., Bielsko-Biała, the top of Kubalonka mountain, 790 m, on the bedding in the spruce wood, 22 Sep. 1986, *K*. *Jędrzejko*, *J*. *Żarnowiec*, *H*. *Klama,* MO513738; Silesian Upland, Garb Tarnogórski, Kanał Baba along the brod-gauged railway, 500 m, SSE of the south-eastern corner of the sediment trap of the Olkusz mine and 300 m N of Szlak Pustynny in Pustynia Starczynowska, 19°30’28”-19°30’39”E, 50°16’16”-50°16’19”N, 324 m, on soil in pine and spruce forest, 18 Sep. 2021, *H*. *Bednarek*-*Ochyra*, *R*. *Ochyra*, *B*. *Godzik 2407/12*, MO6494321; Tatra Mountains, Głodówka, spruce forest, heaps, 05 Aug. 1997, *K*. *Grodzińska*, *B*. *Godzik*, *R*. *Ochyra*, MO5135852; Western Beskid Mts., Silesian Beskid Mts., on the northern slope of Mt. Dębowice S of Bielsko-biała, 670 m, on ground in shady spruce forest , 01. Sep. 1985, *R*. *Ochyra 791*, MO3652864; West Carpathians, Beskid Żywiecki Mts., Przełęcz Krowiarki pass, 980 m, in forest dominated with Norway spruce, *Abies alba*  in admixture, 49°35’18’’N, 19°35’12”E, Aug. 2005, *B*. *Godzik*, *B*. *Cykowska*, MO6494320, Rów Podtatrzański, Głodówka, 1080 m, in forest dominated with Norway spruce, 42°17’42’’N, 20°06’55’’E, Aug. 2005, *B*. *Godzik*, *B*. *Cykowska*, MO6494319. **Russia**: Chabarovsk, reservatum Chechtziri, Magni ad decline boreale jugl Chechupr, 14 Aug. 1981, *V*. *Ya*. *Czerdanzeva*, VLA; 22 Aug. 1984, *I*.*I*. *Abramow*, C-M-34539. **Slovakia**: Montes Malá Fatra, in terra silvatica montis Minčol, 1180 m, Jul. 1949, *Z*. *Pilous*, C-M-34542. **Sweden**: Ångermanland, Säbrå, E slope of Finsviksberget, 62°41,053’N, 17°57,857’E, humid forest ground, 09 Sep. 2013, *L*. *Hedenäs*, *G*. *Odelvbik*, *K*. *Rönblom*, H235485; Göteborg, Stora Torp, på berg, May 1918, *E*. *Hjertman*, H3112236; Nåleskov ved Klåveröd, 27 Sep. 1969, *J*. *Lewinsky*, C-M-34550; Klåveröd, Granplantage, 10 Sep. 1969, *K*. *Damsholt*, *J*. *Lewinsky*, C-M-34551; Västergötl, billihgen, Häggum på gronvötter, Dec. 1929, *A*. *Hülphers*, H311229; Vstm, Ljusnarsberg, Finn-fall, Jämmerdalen, in *Picea*-*Pinus* forest on a hill, on soil, 17 Aug. 1966, *T*. *Koponen*, H3112232.

***Plagiothecium decursivifolium* Kindb. *in* Macoun & Kindb.**

**Austria**: Südlich vom Kehrbach, 850 m, auf Moorwaldboden, 04 Jun. 1938, *J*. *Froehlich,* MO3666798. **Belgium**: Aigret, Fougues, 19 Jun. 1904, *X*. *Mansion*, PC0784936. **Canada**: Britsh Columbia, Mill Creek, Graham I., Queen Charlotte Is., under shade of rotten log, 18 May 1961, *W*.*B*. *Schofield 14121*, ACC B242222, N of Avalon Pennisula, Deep Bight., 48°06’N, 53°57’W, shaded cliff, 01 Jun 2002, *W*.*B*. *Schofield 119525*, *O*. *Lee*, ACC B189939. **China**: Prov. Sichuan, Mount Omei, 26 Aug. 1980, 1500–3000 m, *P*.*H*. *Raven*, *T*.*E*. *Raven*, *C*.*J*. *Chen*, MO2852110. **Czech Republic**: Böhmen, Zwickau, am Grunde von Fichten und Fichtenwurzeln, 50 m, unter dem Mühlstein, 03 Sep. 1904, *V*. *Schiffner*, MO3974492. **Denmark**: Dragør Kommune, open beech woods near small pond in NE park of Kongelunden, Amager, at base of beech, 06 Mar. 2020,*J*.*T*. *Wynns 1939*, C-M-10515; Furesø kommune, Bregenrød Mose, along old railroad track W of Midtervej, S of Grusgravvej, Ravnsholt Skov. Sjaelland, accessed from parking area on Skovomosen, across from Sondre hus, on soil, 08 Jun. 2010, *J*.*T*. *Wynns 2131*, *N*. *Jacobsen*; Sjaelland, Boserup Skov bei Roskilde, am fusse einer Fichte, 02 Oct. 1887, *C*. *Jansen*, MO2563542. **Finland**: North Karjala, Tohmajärvi, Akkala, NE rockfaces of Hiddenvaara, 06 Aug. 1972, *J*. *Lewinsky,* herbarium; Prov. Uusimaa, Sipoo, Immersby, SW of Helgträsk, 30–60 m, southern boreal forest, decaying stump, 26 Jul. 1975, *A*.*J*. *Sharp*, *H*. *Suzuki*, *H*. *Ando 26402*, MO4435880. **France**: Central France, Foret de Murat, prés du ruisseau de Chambeuil, humus sous résineux, 1380 m, 19 Jul. 1980, *P*.*B*. *Pierrot 80216*, PC0784955; Haute-Loire, Saint-Julin-Molhesabate, pentes boisées de la cote 1369, a terre, 1200 m, 28 Jul. 1953, *P*. *Cuynet*, PC0784974, PC0784950, PC0784959, pentes nord de la cote 1359, prés du Felletin, bois résineux, sur l’humus, 1200 m, 28 Jul. 1953, *P*. *Cuynet*, PC0784979; Languedoc-Roussillon, Lozére, les Cubierettes, Mont Lozére, 1480 m, 20 Sep. 2021, *J*. *Bardat 866*, PC0762301; Loire, Massif du Pilat, la Versanne, sous-bois de cote, rochers, 1200 m, 12 Jun. 1954, *P*. *Cuynet*, PC0784968, Rochetaillée gorges du Furan, sous-bois résineux sur l’humus, 800 m, 22 Jul. 1950, *P*. *Cuynet*, PC0784951, sous-bois résineux des nord-est, a terre, 1300 m, 17 Oct. 1954, *P*. *Cuynet*, PC0784953, les Grands-Bois, chemin des Trois-Croix au Beesat, sur l’humus, 1200 m, 23 Jul. 1950, *P*. *Cuynet*, PC0784977, gorges du Furan, sur l’humus, 700 m, 22 Jul. 1950, *P*. *Cuynet*, PC0784972, PC0784963; Puy-de-Dome, Chambon-sur-Lac, pentes boisées au S.O. du Lac., 16. Jul. 1961, *R*.*B*.*R*. *61*.*231*, PC0784980.**Germany**: Baden-Württemburg, deep mossy forest with ice holes and acidic boulders on the S side of the St. Wilhelm River, just SW of Klifermattshof on Feldbergstrasse (K4959), just beyond park area, Naturpark Südschwarzwald, 869 m, 47°53’08.1”N, 07°58’35.8”E, 23 Apr. 2010, *G*. *Rothero*, *J*.*T*. *Wynns*, C-M-10621; Fichtelgebirge, Bischofsgrün, unterhalb der Weissmannfelsen, 800 m, 25 Sep. 1910, *W*. *Krieger*, MO3974493; Hessen, Laubach, unter fichten auf dem Gaulskopfe, 300 m, Nov. 1900, *G*. *Roth*, H3112274; Saxonia, Jul. 1916, *A. Kopsch*, BM14148090; Simonskall, boden der Fichtenwälder auf verwesenden Nadeln, 400 m, 05 Sep. 1931, *E*. *Bartling 10247*, PC0784961; North Rhine-Westphalia, bei Brenscheid südl, Hohenlina, Flesen an der Strasse von Hohenlimburg-Nahmer en Kuunh der Nalonertal, 265–330 m, *E*. *Hegewald*, *P*. *Hegewald 3025*, MO5240603; Redelings, Die Haard, westl. Von Ahsen, 51°42’N, 07°19’E, *E*. *Hegewald*, *P*. *Hegewald 3194*, MO5240612; Rheinland-Pfalz, Kreis, Kaiserslautern, Pfälzerwald westlich von Landstuhl, Morsches Holz, schattig feucht, 300 m, 09. Aug. 1978, *H*. *Lauer*, MO5218886; Süddeutschland, Keris Göppingen, im Fichtewald im Bürgerhölzle, NE von Göppingen, 390 m, 04. Nov. 1973, *Schumm*, H3112296. **Hungary**: Pest, in silvis in decl. Merid. Montis Nagyszénas prope pag. Nagykovacsi, 04 Aug. 1956, *L*. *Vajda*, H3112279. **Latvia**: Livland, Kreis Riga, am Grunde einer Brike im Graben des Weges von Grawenheide nach Potenfeld, River Dreilingsbusch, 24 Aug. 1908, *J*. *Mikutowicz*, BM14148086. **Netherlands**: Hoge Veluwe N. of Arnhem, 10 May 1969, *K*. *Holmen*, *J*. *Lewinsky*, C-M-34521, C-M-34531, C-M 34533; De Steeg, National Park near Arhem, 52°01’N, 06°05’E, 11 May 1969, *K*. *Holmen*, *J*. *Lewinsky*, C-M-34532. **Poland**: Central Poland, prov. Łódzkie, surroundings of Wiśniowa Góra, mixed forest, soil, 16 Apr. 2020, *G*.*J*. *Wolski 123*, LOD15041, Twarda reserve, mixed forest, *Betula pendula* up to 50 cm, 02 May 2019, *G*.*J*. *Wolski 159*, LOD1540, Swolszewice Małe, mixed forest, Quercus sp. up to 50 cm, 26 Apr. 2020, *G*.*J*. *Wolski 127*, LOD1539, surroundings of Tomaszów Mazowiecki city, mixed forest, *Betula pendula* up to 50 cm, 18 May 2020, *G*.*J*. *Wolski 165*, LOD1538, surroundings of Wiączyń reserve, soil, *G. J. Wolski 198*, LOD15037, surroundings of Natura 2000 area Łąki Ciebłowickie”, riparian forest, soil, 17 Apr. 2021, *G*.*J*. *Wolski 375*, LOD15036; prov. Małopolskie, Ochotnica Dolna, trail on Lubań, in beech forest, soil, 02 May 2018, *G*.*J*. *Wolski 39*, LOD15031, Gorce Mts., Obidowa, on ground in spruce forest, 05 Aug. 1973, *R*. *Ochyra 1612*, MO255072, Tatra Mountains, Głodówka, spruce forest, heaps, 05 Aug. 1997, *K*. *Grodzińska*, *B*. *Grodzik*, *R*. *Ochyra*, MO5135840; prov. Podlaskie, Koryciny, „Ziołowy zakątek”, soil, 11 Jul. 2018, *G*.*J*. *Wolski 128*, LOD15030; prov. Pomorskie, Bory Tucholskie, surroundings of Szpitalne Lake, mixed forest, *Pinus sylvestris* up to 50 cm, 08 Aug. 2020, *G*.*J*. *Wolski 243*, LOD15032, pine monoculture, humus, 05 Aug. 2020, *G*.*J*. *Wolski 262*, LOD15034, surroundings of Dolina rzeki Brdy reserve, mixed forest, slope, *Pinus syvestris* up to 50 cm, 08 Aug. 2020, *G*.*J*. *Wolski 249*, LOD15035; prov. Śląskie, Western Carpatians, Silesian Beskid, Barania Góra nature reserve, Wisła (prov. Bielsko-Biała), in mountain spruce forest on mouldering log of *Betula verrucosa*, which is lying in a very wet place, 27 Jul. 1985, *K*. *Jędrzejkow*, *J*. *Żarnowiec*, *H*. *Klama*, MO5137193. **Slovakia**: Montes Mala Fatra, in terra silvatica montis Minčol, 1180 m, Jul. 1949, *Z*. *Pilous*, H3112273; Slovak Paradise, SE of Poprad, Podlesok National Park, *Fagus sylvatica-Picea abies*, mixed forest on plateau (calcareous bedrock) with *Abies alba*, *Acer pseudoplatanus*, *Larix decidous*, *Corylus avbelana*, 800–885 m, 48°56’N, 20°22’00”E, 27 Aug. 1996, *S*. *Huttunen*, *H*. *Jalkanen 432*, H 3243237. **Sweden**: Sörmland, Utö, siliceous rock opposite, L. Ängsholmen, 27 Jul. 1965, *E*. *Nyholm*, MO2154968; Skåne, Högestand, Lyckås, 25 Jul. 1996, *S*. *Medelius*, H3112212; Dalsland, Mo, Öjersbyn, 24 Sep. 1916, *P*.*A*. *Larson*, H3112213, Västergötl, Billingen Berg, Sep. 1933, *A*. *Hülphers*, H3112220, Skåne, Högertad, Lyckås, 25 Jul. 1916, *S*. *Medelius*, H3112210; Tore Lappmark, Karesuando, W of Tsuonamavaara, 470 m, 68,233568°N, 22,242581°E, rock crevice, 31 Aug. 2019, *L*. *Hedenäs*, *A*. *Jörgensen*, *G*. *Odelvik*, *M*. *Westberg*, *U*. *Swenson*, MO6961016; Västmanland, Viker s.n., Älvlånga Lake, 16 Aug., *K*. *Holmen*, MO2500267; Skåne, Veberöds, Kvarnbrodda, 19 Sep. 1923, *O*.*J*. *Hasslow*, herbarium. **Switzerland**: Kanton Bern, Burgfeld ob. Beatenberg, auf Tannenwurzeln in der Nähe der oberen Waldgrenze, 1630–1700 m, 31 Jul. 1912, *P*. *Culm,* MO3974490.

***Plagiothecium imbricatum* G.J. Wolski & W.R. Buck**

**Canada**: Britsh Columbia, Alice Ridge, trial to Diamond Head Mt., base of tree in forest, 49°35’N, 123°05’W, 29 Sep. 1983, *W*.*B*. *Schofield*, *J*. *R*. *Spence 81577*, ACC B58228. **Great Britain**: *J*.*G*. *Gardiner*, BM13777470. **Netherlands**: Hoge Veluwe N. of Arnhem, 10 May 1969, *K*. *Holmen*, *J*. *Lewinsky*, C-M-34530.**Poland**: prov. Łódzkie, surroundings of Wiączyń reserve, humus at the drainage ditch, 01 Jun. 2020, *G*.*J*. *Wolski 190*, LOD15043, beech forest, humus, 01 Jun. 2020, *G*.*J*. *Wolski 202*, LOD15044, soil, 01 Jun. 2020, *G*.*J*. *Wolski 191*, LOD15042, Lublinek forest, mixed forest, soil, 07 May 2020, *G*.*J*. *Wolski 118*, LOD15046; prov. Kujawsko-Pomorskie, surroundings of Dolina rzeki Brdy reserve, slope near the river on soil in mixed forest, 13 July 2020, *G*.*J*. *Wolski 424*, LOD 15015, NY04688394, SZUB-B 00001.
